# Improved Safety of Nucleic Acid Amplification Technology Combined With Serological Tests for Screening Blood Donors: A Systematic Review and Meta‐Analysis

**DOI:** 10.1002/rmv.70117

**Published:** 2026-02-21

**Authors:** Heloise Skiavine Madeira, Aline Ávila Brustolin, Marcos Elias da Silva Almeida, Maiara Vanusa Guedes Ribeiro, Raul Gomes Aguera, Fernando Américo Jorge, Luciana Dias Ghiraldi, Débora de Castro Moreira, Maria Valdrinez Campana Lonardoni, Dennis Armando Bertolini, Leyde Daiane de Peder, Claudinei Mesquita da Silva, Daniele Stéfanie Sara Lopes Lera‐Nonose, Áquila Carolina Fernandes Herculano Ramos‐Milaré, Jorge Juarez Vieira Teixeira

**Affiliations:** ^1^ State University of Maringa (UEM) Maringá Paraná Brazil; ^2^ Assis Gurgacz Foundation University Center Cascavel Paraná Brazil

**Keywords:** blood donors, hepatitis B virus, hepatitis C, nucleic acid amplification techniques, serologic tests

## Abstract

No laboratory test performed to date provides an absolute guarantee for detecting infectious agents. Nucleic acid amplification techniques/tests (NAT) associated with serological tests can increase safety and provide greater diagnostic accuracy for patients. We investigated the added safety of NAT technology combined with serological tests for screening blood donors for Hepatitis C virus (HCV), human immunodeficiency virus (HIV) and hepatitis B virus (HBV). Data were obtained from a systematic search conducted up until march 30, 2024 in five electronic databases: *PubMed, Web of Science, Scopus, Embase*, and the *Cochrane library*. Twenty‐nine studies, published between 1998 and 2023, were included in the review. Notably, HBV infection was predominant among donors with positive NAT results, with 425 cases, including 373 with positive serology and 52 with negative serology. Data from the diagnostic window period (WP) indicated the highest number of HBV infection cases, with 154 reported. Of the 29 included studies, 10 reported cases of occult HBV infection and diagnostic WP infection. Thirteen studies identified donors during the occult HBV infection period, and 17 identified WP cases. Meta‐analyses of positive NAT results following negative serological screening for HBV, HCV, and HIV revealed a significantly pooled frequency (*p* < 0.001). Positive NAT results retrieved from donors with negative serological HBV and HCV showed a significantly combined frequency (*p* < 0.001), while for HIV occurs the opposite (*p* = 0.085). For HBV, HBC and HIV serological tests and NAT‐positive results, the pooled frequency was significantly (*p* < 0.001). The findings demonstrate the positive relationship and safety of molecular technology in screening blood donors for HBV, HCV, and HIV using serological tests. Molecular testing remains a valuable tool for detecting and elucidating bloodborne infections.

**Abbreviations**
Ab: Antibody
Ag: Antigen
ALT: Alanine Aminotransferase Enzyme
CI: Confidence Interval
CLIA: Chemiluminescence Immunoassay Methodology
CMIA: Chemiluminescence Assays
ECLIA: Electrochemiluminescence Immunoassay
EIA: Enzyme Immunoassay
ELISA: Enzyme‐Linked Immunosorbent Assay
ES: Effect Size
HBC: Antibody Against Viral Core Antigen of HBV
HBC: Anti‐hepatitis B Core Antigen
HBEAG: Hepatitis B Virus E Antigen
HBSAG: Hepatitis B Virus Surface Antigen
HBV: Hepatitis B Virus
HCV: Hepatitis C Virus
HIV: Human Immunodeficiency Virus
HP: High Prevalence
ID‐NAT: Individual Donation Nucleic Acid Test
LOD: Limit of Detection
LP: Low Prevalence
MEIA: Microparticle Enzyme Immunoassays
MP‐NAT: Mini‐pool Nucleic Acid Test Multiplex Assay
MPX: Multiplex
NAT: Nucleic Acid Amplification Techniques
NATPOS: Positive NAT
NR: Not Reported
OBI: Occult Hepatitis B Infection
PCR: Qualitative Polymerase Chain Reaction
PRISMA: Preferred Reporting Items for Systematic Reviews and Meta‐analyses
qPCR: Real Time Quantitative Polymerase Chain Reaction
RT‐PCR: Reverse Transcriptase–polymerase Chain Reaction
S. E. Of. Logor: Standard Error of the Logarithm of the Odds Ratio
SERONDONORS: Negative Donors Serological Tests
SERONNATP: Serology Negative and Positive NAT
SEROPNATPHBV: Serology and NAT Positive for HBV
TMA: Transcription Mediated Amplification
TTIS: Transfusion‐transmitted Infections
VL: Viral Load
WP: Window Period

## 1 Introduction

Blood transfusion is a common medical procedure. Although generally safe, it carries inherent risks, as blood is a living biological fluid capable of transmitting infectious diseases. Several infections, especially those caused by viruses, can be transmitted through blood transfusion. Ensuring transfusion safety remains a major global challenge, as transfusion‐transmitted infections continue to pose a residual risk despite advances in donor screening and laboratory detection of infectious agents. Addressing this residual risk remains a central focus of hemotherapy research [1].

In recent years, Brazil registered a Blood transfusions increased from 3,088,332 in 2023 to 3,178,138 in 2024, a growth of 2.9% [2]. Serological screening techniques used for blood donor testing demonstrate sensitivity and specificity for established infections; however, these methods are limited by the immunological window period (WP), during which recently infected individuals may harbour transmissible levels of viraemia in the absence of detectable serological markers [3, 4, 5].

The introduction of nucleic acid amplification technology (NAT) represented a major advance in transfusion safety by enabling direct detection of viral genetic material independently of the host immune response. Since its initial implementation in the 1990s, NAT has substantially improved the detection of human immunodeficiency virus (HIV), hepatitis C virus (HCV), and hepatitis B virus (HBV) [6, 7].

Observational studies indicate that NAT identifies potentially infectious donations missed by serological testing alone (NAT yield), particularly during the immunological WP, resulting in measurable reductions in transfusion‐transmission risk, especially for HIV and HCV [8, 9]. A large international survey across 32 countries reported over 3100 NAT‐positive donations in 2019, highlighting the global impact of NAT on blood safety [7].

Despite advances and expanded use of NAT for molecular surveillance and emerging pathogen screening, [7] its optimal role within blood donor screening algorithms remains debated, particularly regarding the added value of combining NAT with serology. While residual risks for HIV and HCV are now extremely low in many settings, HBV remains challenging due to occult infection, low‐level viraemia, and variability in NAT analytical sensitivity, which complicate residual risk estimation [10, 11, 12].

Evidence indicates that NAT and serological testing play complementary roles in donor screening. While serological assays are particularly effective for detecting established infections, especially among first‐time donors, NAT performs better in identifying WP infections and infections in repeat donors, in whom seroconversion may not yet be detectable [3, 11].

The limited number of scientific publications validating both diagnostic techniques highlights the need for further research. To our knowledge, this is the first meta‐analysis investigated on the topic in question. We investigated the safety of NAT technology combined with serological tests for screening blood donors for hepatitis C virus (HCV), human immunodeficiency virus (HIV), and hepatitis B virus (HBV) through a systematic review and meta‐analyses.

## 2 Method

### 2.1 Protocol and Registration

The research was conducted according to the Preferred Reporting Items for Systematic Reviews and Meta‐Analyses (PRISMA) protocol [13, 14], and checklist (Supporting Information S1: File 1) including registration in the PROSPERO platform (CRD 42021273094).

### 2.2 Research Strategy

The research question was organised according to the *PICO* strategy, using the acronym: *Participants:* blood donors; *Intervention*: NAT and serological assays; *Comparison:* n/a; *Outcome:* confirmatory or non‐confirmatory serological tests and NAT for HBV, HCV, and HIV. Does the Nucleic Acid Amplification Techniques/NAT associated with serological tests to detect blood donors with HBV/HCV/HIV in the WP or in the presence of occult infections increase donor safety?

The research initially focused on classifying the research descriptors by seven researchers in Group 1 (HSM, MESA, MVGR, RGA, FAJ, LDG, DCM). Two researchers with extensive experience reviewed and validated the MeSh term blocks (JJVT, MVCL) (Supporting Information S2: File 2). Five databases PubMed, Web of Science, Scopus, Embase, and the Cochrane library were used to retrieve data from articles published by March 30, 2024. The PubMed search followed the (MeSH) strategy, while subject, titles, keywords, and abstract were used in Web of Science and Scopus. For each database, the terms were organised into three blocks and related to each other. The first block for PubMed, Web of Science, Scopus, and the Cochrane library contained the terms, block 1, ‘nucleic acid amplification techniques’, ‘serologic tests’, block 2 ‘blood donors’, ‘blood donation’, and block 3 ‘hepatitis C’, ‘hepatitis B virus’, ‘hepacivirus’, ‘hepatitis B’, and ‘HIV’. In the Embase database, an advanced search was performed using terms such as ‘nucleic acid amplification techniques’, ‘serologic tests’, ‘blood donor’, ‘blood donation’, ‘hepatitis B’, ‘hepatitis C’, ‘human immunodeficiency virus’, ‘hepatitis B virus’, and ‘hepacivirus’. We used the Boolean operator OR to increase sensitivity between the terms and to associate the three blocks. To enhance search results, we performed a free‐term search (ti = title) in the databases. We incorporated all MeSH terms from the 3 search blocks and two other relevant terms (nucleic acid amplification test or nucleic acid amplification technology) to broaden the search. Filters were applied for the English language, human participants, and available abstracts, regardless of the published period.

### 2.3 Criteria for Selection of Articles

Based on the titles and abstracts, the researchers in Group 1 selected articles that aligned with the study objective by consensus. Comments, reviews, editorials, letters, case reports, interviews, news reports, guidelines, errata, conference abstracts, and studies comparing molecular techniques and cost effectiveness were excluded.

Articles were retrieved in full‐text PDF format, organised and structured into blocks, and randomly distributed to the researchers of Group 1 for independent review. The group's researchers used consensus methods to resolve any doubts. References were checked for the selected articles to recover publications not selected during the initial stage.

### 2.4 Data Extraction

For data extraction, 29 studies were selected. Relevant information was extracted from each article, including the author, country, place of research, study period, objective, described infection (HBV and/or HCV and/or HIV), number of donors on whom tests were performed, methods used in NAT, serology performed, study limitations, and statistical analysis. Additional information, such as the performance of main confirmatory tests, the number of cases of occult infection, diagnostic WP, residual risk, results, and a brief conclusion, was also extracted. Several consensus meetings were held to analyse the final structure of the tables and define the most relevant results of the search. Finally, for independent validation of the selected publications and tables, Group 2, comprising six judges (MVCL, DAB, LDP, CMS, DSSLL‐N, ACFHR‐M), was organised.

### 2.5 Assessment of Methodological Quality

Initially, all researchers individually analysed the publications regarding limitations or risks of bias reported in the selected articles. In the second stage, methodological quality was analysed using the tool for assessing diagnostic accuracy (QUADAS‐2) [15]. The completion of a standard questionnaire was conducted independently by four experts (JJVT, MVCL, DSSLL‐N, and ACFHR‐M). After individual analyses, each researcher resolved discrepancies through consensus with another randomly selected specialist. Four domains were assessed for risk of bias: (1) patient selection, (2) index test, (3) benchmark test, and (4) flow and timing, while three domains (1, 2, and 3) were evaluated for applicability concerns.

### 2.6 Statistical Analysis

Meta‐analyses was performed using the STATA programme (version 12, Stata Corporation, College Station, TX, USA). The odds ratio was used to estimate the concordant proportions between the laboratory tests in the publications, with a 95% confidence interval (CI). A subgroup analysis was undertaken to investigate the consistency of results, comprising the number of serological tests and NAT provided by each publication. The values of the proportions between the tests (serological and NAT, positive or negative) enabled the elaboration of the forest plot, showing the individual measures of each publication and the aggregated measure. To calculate heterogeneity and inconsistency between publications, Cochran's Q test (*p* < 0.10) and the criteria proposed by Higgins and Thompson (I^2^), namely low, moderate, and high (25%, 50%, and 75%) were used [16]. We employed Begg's rank correlation test [17] and Egger's linear regression test [18] to assess publication bias, with significance set at *p* < 0.05. Pooled estimates were calculated according to the random effects model [19].

## 3 Results

**FIGURE 1:**
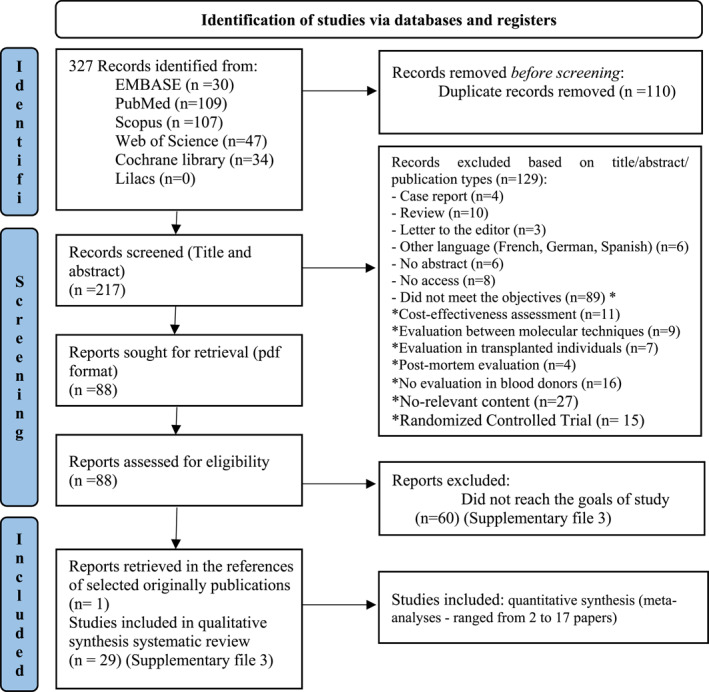
Flowchart indicating the number of findings per database, and the detailed selection process until the inclusion of the final 29 studies, as suggested by the PRISMA 2020 guidelines.

The search strategy in the six databases identified 327 records. After screening, 28 publications were included in the final sample. One article was recovered from the references, totalling 29 articles for the systematic review, with a range of 2–17 articles included in the meta‐analyses. The articles included in the meta‐analysis must adhere to the close relationship between serological tests and NAT described in Sections 3.2.1 to 3.2.3 (Figure 1).

The selected studies were published between 1998 and 2023; the data period covered was from July 1997 to June 1980 and from January 1999 to November 2022, with sample sizes ranging from 50 to 46 million donor candidates. The Asian continent was the predominant study location [20, 21, 22, 23, 24, 25, 26, 27, 28, 29, 30, 31, 32], followed by Europe [33, 34, 35, 36, 37, 38, 39, 40]. Screening for all three infections was observed in most studies [20, 21, 22, 24, 25, 26, 28, 32, 33, 34, 41, 42, 43, 44, 45]. Only one study screened for both HCV and HIV [35]. Regarding individual screening, nine studies screened for HBV [23, 27, 29, 31, 35, 37, 40, 46, 47] four studies for HCV [30, 37, 38, 47] and only one study screened for HIV [48].

The kits employed varied for both NAT and serology methodologies. For NAT, seven studies used only transcription‐mediated amplification (TMA) [20, 32, 34, 35, 38, 42, 46] while 22 articles [21, 22, 23, 24, 25, 26, 27, 28, 29, 30, 31, 33, 36, 37, 39, 40, 41, 43, 44, 45, 47, 48] used the polymerase chain reaction (PCR) technique to detect and analyse specific viral DNA/RNA sequences. Among the 22 studies, six [22, 23, 31, 33, 40, 41] performed tests with both molecular techniques (PCR and TMA). In serology, several serological markers were evaluated, depending on the infection and specific objectives of each study.

Regarding statistical analysis, although most studies did not report which specific test was used, mentioning only the programme, 13 studies conducted analysis comparing viral loads of reactive samples using serological markers [23, 25, 35, 39, 44] and estimating the residual risk [25, 34, 38, 41, 43], yield of NAT [32], and sensitivity and specificity of the assays [21, 28]. Seven studies described the limitations, among these, some did not state the proportion of NAT yield cases for HBV representing occult infection due to the lack of anti‐HBc serological marker analysis, and the impossibility of following donors to observe seroconversion [24] others reported that the analysed data did not cover all anti‐HBc screened donations during the observation period [39]. Furthermore, limitations regarding the availability of commercial HIV NAT assay kits capable of detecting only HIV‐1,^26^ possible software errors and handling mistakes [21, 45], sample size [30, 36], and reagent values in serology for certain infections were underreported, as indeterminate serology results were not collected and, after confirmation, more donors may have tested positive for reagents [44]. These factors hindered the identification of differences in the studied variables (Table 1).

**TABLE 1:** Characteristics of included studies in the systematic review and meta‐analysis.

Study country	Period of study	Infection (HIV, HCV, HBV)	Number of donor	Method		Results	Conclusion
				NAT method	Serological assay		
Kosan et al. [20] Turkey	February 2007 to March 2008	HIV (1 and 2), HBV, and HCV	18.200	Discrimination of HIV, HCV and HBV: Procleix Ultrio kit (chiron ltd., USA), based TMA (transcription mediated amplification) for the NAT study.	Test for anti‐HIV/1–2, anti‐HCV, HBsAg: – Vironostika HIV Uni‐Form II Ag/Ab; Innotest HCV Ab III; Hepanostika Ultra HBsAg; Serology (+) NAT (−): – anti‐HBc test; anti‐HBe test; Confirm the anti‐HIV and anti‐HCV tests: – Immuno‐blot test; Serology (−) NAT (+): – Micro‐ELISA (in the macro‐ELISA Axim instrument).	Serological methods: – anti‐HIV/1–2 positive: 24 (0.13%); – anti‐HCV positive: 66 (0.36%); – HBsAg positive: 318 (1.75%). NAT discrimination test: – HIV/1 positive: 4 (0.02%); – HCV positive: 12 (0.06%); – HBV‐DNA positive: 312 (1.72%). Serology (+) NAT (+) combination: – HIV positive: 3; – HCV positive: 9; – HBV positive: 297. Serology (−) NAT (+) combination: – HCV positive: 2; – HBV positive: 11.	The number of serologic‐negative and NAT positive donors was higher than those of other studies and a viral risk (HBV, HCV) was found in 1 of 1400 transfusions. If consider the high costs of NAT testing compared with other serologic tests, NAT was not found to be efficient in all studies on a cost‐based effectiveness. Therefore, NAT should be applicable only for first time donors at regional blood centres.
Li et al. [21] China	July 30 to September 17, 2006	HIV (1 and 2), HBV, and HCV	10.824	Test for HBV, HCV, HIV‐1: COBAS AmpliScreen tests—PCR.	Routinely screened: – HBsAg; anti‐HCV; anti‐HIV‐1/2; Confirmation tests: – HBsAg neutralisation test; anti‐HCV recombinant immunoblot assay; anti‐HIV‐1/2 western blot; Supplemental tests: – anti‐HBs; anti‐HBc IgG; anti‐HBc IgM; anti‐HCV (AxSYM, Abbott diagnostics); anti‐HIV combo (AxSYM, Abbott diagnostics); Samples with discordant results: – HBsAg with the PRISM (Seattle); anti‐HCV (PRISM, in Hong Kong).	The 95% limit of detection (LOD): – HBV 5.09; – HCV 11.83; – HIV 62.53 IU/mL. Seronegative donations (10,727): – HBV NAT 12 (0.11%); – HCV NAT 1 (0.01%). Follow‐up results for 1–8 months: – HCV yield case was a window case; – All HBV NAT yield cases were occult carriers; This study showed that approximately 15.8% (11/70) of HBsAg‐positive donations were missed by MP‐NAT.	The use of NAT detected occult HBV and reduced HCV window period. The yield rate, especially occult HBV, was 10 to 100‐fold higher than that in developed, HBV nonendemic countries. Therefore, NAT implementation for routine donor screening in a more cost‐effective manner should contribute to safer blood transfusion in Taiwan.
Lin et al. [22] China	NR	HBV, HCV and HIV	5.973	Simultaneous detection and discrimination of HIV RNA, HCV RNA, HBV DNA: MPX v2 test (roche molecular systems, USA) and ultrio test (novartis diagnostics, USA). ultrio test (novartis diagnostics, USA). Reactive samples: Procleix discriminatory HBV, HCV and HIV‐ 1 tests.	Initial screening: – HBsAg; anti‐HCV; anti‐HIV‐1/2; HBV positive – Vitros Immunodiagnostic test; Abbott AxSYM HBV 3.0 Anti‐HCV‐positive; – Vitros Immunodiagnostic test; Abbott AxSYM HCV 3.0 Anti‐HIV‐positive; HIV BLOT 2.2. – Additional serology testing was carried out which included total anti‐HBc, anti‐HBc IgG, anti‐HBc IgM, anti‐HBs, HBe and anti‐HBe.	Eight donors were confirmed serology RR: – Six HBV and two HCV. All six HBV‐infected donors were also HBV NAT reactive, and one of the two HCV confirmed serology RR donors was NAT reactive. The follow‐up period varied from 35 to 8 months: All 8 donors had an occult HBV infection with viral loads < 12 IU/mL.	The introduction of the MPX v2 test in regions with a high prevalence of HBV would add an extra layer of safety to the blood supply by interdicting samples from donors with an occult HBV infection.
LouisirirotchaNakul et al. [23] Thailand	NR	HBV	175	Simultaneous detection ‐ HBV, HCV, and HIV‐1: PROCLEIX ULTRIO test (ultrio test). Additional detection of HIV‐1 groups O and HIV‐2: Roche cobas TaqScreen MPX test.	Immunologic screening of index blood donations: – PRISM HBsAg; anti‐HCV; ARCHITECT anti‐HCV, anti‐HIV (Abbott). HBV NAT–reactive/HBsAg‐negative index samples: – HBsAg; anti‐HBs; total anti‐HBc; HBeAg; anti‐HBe.	The 175 HBV NAT–reactive/HBsAg‐negative index samples were further investigated. Of the donors with an acute HBV infection: − 15 were in the HBV window period; − 4 were in the late stage of acute infection. The majority of donors (48/72 or 66.7%) had an occult HBV infection. There were three breakthrough infections (or reinfections).	The majority of donors detected during routine screening, who were HBsAg negative and NAT reactive, had an occult HBV infection, thus validating the decision to introduce NAT for blood donations in Thailand.
Moiz et al. [24] Pakistan	April 2011 to november, 2012	HBV, HCV and HIV‐1	42.830	Screening for HBV DNA, HCV, HIV‐1 (group M, O), and HIV‐2 RNA: Donor samples were tested in pools of six and resolution of positive pools was performed individually, by a multiplex PCR test (cobas Taq screen MPX, roche).	Tests: Anti‐HIV 1/2; Anti‐HCV and HBsAg; Routinely screened: – Third‐generation automated chemiluminescence immunoassay (CLIA) analyser; Confirmation tests: – Enzyme‐linked immunosorbent assay–positive samples by HIV‐1 western blotting.	There were 1526 (or 3.5%) donors having 1571 abnormal screening results with 45 who were serologically reactive to two or more disease markers: – HCV antibodies (anti‐HCV; *n* = 708); – HBsAg (*n* = 555); HIV antibodies (anti‐HIV; *n* = 29). 35 NAT‐reactive samples were identified: – One HIV‐1; − 27 (55%) HCV; − 7 (14%) HBV.	The current risk of transfusion transmitted viral infections attributable to blood donation is relatively high in this country. The study recommends the parallel use of both serology and NAT screening of donated blood in countries that have high seroprevalence of these viral infections.
Niazi et al. [25] Pakistan	September 21, 2012, to September 20, 2013	HBV, HCV and HIV	56.772	Seronegative blood donor samples: Multiplex polymerase Chain reaction kit, (cobas TaqScreen MPX test).	Serological screening: HBsAg, anti‐HCV, and HIV antigen‐antibody combination assay on an automated immunoassay analyser, (Architect i2000, Abbott).	2334 blood donors were found to be reactive: − 719 (1.27%) were reactive for HBsAg; − 1046 (1.84%) for anti‐HCV; − 12 (0.02%) HIV; − 557 (0.98%) for syphilis antibodies. Between 54,438 seronegative donors: 27 NAT‐reactive donors: − 23 for HBV DNA (HBV NAT yield, 1: 2367); − 4 for HCV RNA (HCV NAT yield, 1: 13,609). No case of HIV was detected by NAT during the study period.	NAT led to interdiction of donations from 23 hepatitis B– and 4 hepatitis C–reactive donors in 1 year, thus improving the safety index of our blood products. NAT has improved the safety of blood products.
Selim et al. [26] Saudi Arabia	January 2009 and June 2011	HBV, HCV and HIV‐1	12.437	RNA or DNA extraction: RT‐PCR cobas AmpliPrep amplification and detection: Cobas Amplicor analyser (roche).	Serologic enzyme immunoassays: HBsAg, HBcAb (total), HCV antibodies, HIVp24Ag/Ab, and HIV‐1 and HIV‐2 antibodies (AxSYM analysers, Abbott, and BEP 2000‐ Siemens).	− 405 seropositive donors. − 12,032 seronegative: NAT 5 (0.042%) were positive for HCV and 2 (0.017%) for HIV‐1. – None seronegative donors were NAT positive for HBV.	The introduction of MP NAT screening for blood donors in blood bank allowed better detection of TTVs, especially for HCV and HIV‐1 among the studied donors.
Yoshikawa et al. [27] Japan	February 1, 2000, to March 31, 2003	HBV	328	RNA or DNA extraction: Cobas AmpliPrep machine (roche). Amplification and detection: Cobas Amplicor analyser (roche).	HBsAg: EIA, CLIA; Anti–HBc: EIA; Haemagglutination inhibition assay ALT: By machinery with reagents of transaminase HR‐II.	328 NAT‐positive donations, 26 were from donors who could be followed‐ up in both increasing and decreasing phases of HBV DNA load. HBsAg was not detected during observation in three donors; 3 OBI with HBV DNA less than 10^5 copies/mL.	The NAT is not only valuable for detection of low HBV DNA levels in the pre‐ and post‐HBsAg window periods, but also may be capable of identifying higher levels of viraemia in anti‐HBc–negative donors with occult HBV infection in either an acute or chronic stage.
Vermeulen et al. [40] south African	October 1, 2005 to September 30, 2006	HBV, HCV and HIV	732.250	ID‐NAT confirmation test: HIV versant bDNA 3.0 viral load assay qPCR (confirmation test).	Serology – Chlia (PRISM Abbott); – EIA to HIV and HCV and neutralisation test for HBsAg; – Versant bDNA 3.0 viral load assay; – Innotest p24 antigen assay.	– HIV positive 738 (720 HIV RNA+/Anti‐HIV+); – HBV positive 515 (435 HBV DNA+/HbsAg+); – HCV positive 39 (29 HCV DNA+/anti‐HCV+). The residual risk obtained in this work was: − 6.98 (1:143.226) to HIV; − 48.45 (1:20.642) to HBV; − 0.061 (1:16.226.411) to HCV.	One‐year ID‐NAT screening of 732,250 donations interdicted 16 HIV, 20 HBV, and 1 HCV window phase donations and 42 anti‐hepatitis B core antigen–reactive infections during an early recovery (6) or a later stage (36) of occult HBV infection.
Barbosa et al. [48] Brazil	NR	HIV‐1	50	PCR	Repeated HIV‐1: – Reactivity in the screening test (ELISA); – Initial indeterminate western blot results.	– Between 50 blood donors selected based on HIV‐1 repeated reactivity on screening test by ELISA and initial indeterminate western blot results, the WB results were 3 positive, 18 negative and 29 indeterminate. – PCR showed amplification of the HIV‐1 provirus DNA in 12 samples. All donors with positive WB (*n* = 3) had also a positive PCR. 4/18 donors with reactive ELISA but negative WB and 5/29 with indeterminate WB were positive in the HIV‐1 PCR. – HMA of the amplified DNA showed that 12 belonged to HIV‐1 B subtype and one to F subtype.	PCR was useful to elucidate a number (5/29) of indeterminate results for HIV‐1 in blood donors. The donor must be monitored closely and cautiously, perhaps in a referral centre.
Cable et al. [41] South Africa	October 2005 to September 2010	HIV, HBV and HCV	649.745	Procleix ultrio multiplex assay and the Tigris analyser. Any seronegative potential HBV NAT yield cases were confirmed by follow‐up and⁄ or viral load testing by quantitative PCR (q‐PCR) ID‐NAT using the ultrio assay on the Tigris instrument (novartis diagnostics).	– Screened for anti‐HIV 1 and anti‐HIV 2, anti‐HCV and HBsAg: Prism analyser (Abbott); – If anti‐HIV is reactive but NAT is nonreactive: Western blot; – If HBsAg, anti‐HBc, IgM anti‐HBc and anti‐HBs reative: IMx Abbott. – Anti‐HCV rective: Enzyme immunoassay (Abbott).	– 249 HIV positive (241 HIV‐RNA+⁄ HIVAb+; 6 HIV‐RNA + WP; 2 elite controllers); – 409 HBV‐positive (371 HBV‐DNA+⁄HBsAg+; 21 all HBV NAT yield; 17HBsAg+⁄HBV‐DNA nonreactive) − 15 HCV‐positive donations. Mathematical modelling estimated – Similar HIV transmission risk for lapsed and repeat donations (3 per million); – Window period risk for HBV was 13 per million; – There were significantly more anti‐HBc‐positive donors in the ultrio initial reactive ⁄ non repeat reactive group (12%) than in an ultrio nonreactive control group (6%).	The introduction of ID‐NAT significantly contributed to increasing blood safety in terms of the risk of HIV and HBV transmission with little impact on the risk of HCV.
Fiedler et al. [32] Germany	2008 to 2015	HIV‐1, HCV, and HBV	46.151.554	NAT: – HIV‐1‐NAT; – HCV‐NAT; – HBV NAT. Additional information: – Type of NAT screening assay; – LoD; pool size; viral load; – Genotype of the respective vírus.	Screened for Anti‐HBc and HbsAg.	Transmissions associated with donations in the viraemic pre seroconversion window period one HIV‐1, no HCV and four HBV.	The currently practiced donor screening strategy ensures a high standard of blood safety.
Kleinman et al. [45] USA	August 2002 to April 2003	HBV	704.902	Minipool nucleic acid testing (MP NAT): COBAS AmpliScreen HBV test (roche molecular systems).	− HBsAg, and HBc (Auszyme e ortho clinical diagnostics); − Anti‐HBc total (EIAs; Abbott laboratories, ortho clinical diagnostics); − Follow‐up tests: Anti‐HBc total and IgM; HBsAg and Anti‐HBs.	− 578,671 specimens were negative; − 84 classified as positive based on concordant positive DNA and serology results; − 16 classified as positive based on HBV serology results; − 3 classified as positive based on concordant positive DNA and HBsAg results; − 4 donors reclassified as HBV status negative based on negative follow‐up testing; − 23: 2 donors classified as HBV‐positive window‐period and 21 donors classified as HBV status–negative.	The data indicate that the implementation of HBV MP NAT would likely cause the interdiction of several HBV WP units and could prevent numerous other cases of transfusion‐transmitted HBV infection annually.
Sgourou et al. [33] Greece	1 January 2005 to 31 March 2007	HBV, HCV and HIV‐1	38.264	− The procleix ultrio TMA qualitative NAT assay system. – Automated quantitative PCR. − COBAS AMPLICOR HBV/HCV monitor test; − Versant HIV RNA 3.0 test.	− HIV/1–2, HCV and HBsAg AxSYM HBsAg (V2) microparticle enzyme immunoassays MEIA—Abbott). − Additional serological assays: anti‐HBc, IgM anti‐HBc, HBeAg, anti‐HBe and HBsAg.	The total prevalence of HBV infection was 1.4% and 0.2% for HCV. Reactive samples: − 45 positive for HBV (42 first time and 3 repeated donors); − 8 for HCV (all first‐time donors); − None for HIV‐1 and none for two or three viruses together. Occult HBV infection (OBI): − 7 donations NAT positive but negative in all serological testing including HBsAg.	The universal implementation of ID NAT for HBV requires further assessment of method sensitivity and testing algorithms in the daily practice of blood transfusion centres in order to evaluate their impact in detection of infections in WP (window period) and of OBI.
Stolz et al. [34] Switzerland	June 2007 to February 2009	HBV	306.000	Procleix ultrio assay on the Tigris system for HIV‐1 RNA, hepatitis HCV RNA, and HBV DNA. Ultrio assay on Tigris system, Gen‐Probe/Novartis).	Enzyme‐linked immunoassays: − Enzygnost HBsAg (dade behring). − Axsym HBsAg V 2.0 assays (Abbott). − Chemiluminescent assay Architect HBsAg (Abbott).	− The prevalence of acute HBV infections was 5 in 269,000. − The prevalence of OBI in all donors was 1:61,000. The mathematical model for calculation of the transmission risk of HBV in WP donors: − If only HBsAg screening had been performed: 1:95,000. − NAT reduced the overall risk 3.1‐fold, to 1:296,000.	NAT alone was more efficacious than the combined use of HBsAg and anti‐HBc. The data from this study led to the decision to introduce sensitive HBV‐NAT screening in Switzerland.
Wang et al. [28] China	June 2010 to March 2012	HBV, HCV and HIV	826.044	MP of six and individual donation. Discriminatory; TaqMan for the cobas TaqScreen MPX test. When reactive, ID nat was performed.	HBsAg‐negative donations were tested further for anti‐HBs and anti‐HBc with an anti‐HBs and anti‐HBc assay.	A total of 826,044 blood donations that were negative for HBsAg, anti‐HCV, anti‐HIV; 413 of these donations were HBV DNA positive. According to supplemental testing for anti‐HBc and anti‐HBs, 17.2% of confirmed HBV DNA–positive donors probably were in the highly infectious window period and 56% were potentially infectious occult HBV carriers because neutralising anti‐HBs antibodies were not detectable.	Approximately 10% of non‐resolved MPs contain HBV DNA from a low‐viral‐load occult carrier. ID‐NAT resolution testing in duplicate to minimise HBV transmission risk associated with transfusing nonreactive donations implicated in reactive MPs.
Yang et al. [29] China	June 2010 to January 2011	HBV	65.800	AmpliPrep/TaqMan HBV v 1.0 test; AmpliPrep/TaqMan HCV v 1.5 test, and COBAS AmpliPrep/TaqMan HIV‐1 TaqScreen MPX test.	HBsAg test, ELISA test and WanTai ELISA test. HBV DNA reactive samples were tested for HBsAg, anti‐HBs, HBeAg, anti‐HBe, and anti‐HBc using the electrochemiluminescence immunoassay with an immunoassay analyser.	A total of 80 NAT test‐reactive pools were identified and 59 pools (74%) resolved to a reactive sample. − 15 samples could not be confirmed as NAT reactive either by an alternative NAT test or by serology. − Six donors were found to be HBsAg positive on retesting the index donations with the ECLIA test. − Three of the donors were nonreactive for HBV DNA, classified as HBV window period donors. The majority of donors (55.9%) had an occult HBV infection, followed by chronic infections (10.2%), window period infections (5.1%), and acute resolving infections (3.4%).	HBV is one of the most prevalent transfusion‐transmitted infectious diseases in China, screening of donations by NAT would be particularly beneficial.
Zhang et al. [30] China	NR	HCV	49 samples from 11 donors	QiaAmp viral RNA kit RT‐PCR assay.	HCV core antigen with an HCV Ag ELISA assay.	27 of 36 (75%) HCV RNA‐positive specimens were HCV core antigen‐positive. 27 (93.1%) of 29 HCV core antigen‐positive samples were found to be HCV RNA‐positive. The average time for HCV RNA to be became detectable was 20.4 days (range, 14–29 days), for HCV core antigen was 23.7 days (range, 14–40 days) and for HCV Ab was 56.5 days (range, 33–74 days).	The HCV core antigen assay is cheap, easily performed, and compatible with equipment now used in blood centres and plasma stations. Moreover, the development of the newer, more‐sensitive quantitative second version of the assay will greatly improve the performance of HCV core antigen assay.
Wu et al. [31] China	August 1, 2010 to December 31, 2019	HBV	1.160.355	TMA, individual NAT for screening and discriminatory assays. HBV NAT yield cases were tested for viral load using the roche cobas AmpliPrep with real‐time polymerase chain reaction performed on a cobas TaqMan analyser.	ECLIA using a cobas e601 analyser or CLIA with an ARCHITECTTM i2000SR analyser (Abbott).	− 476 NAT yields: 19 were probable WP; 33 probable OBIs; 409 were confirmed OBIs; 15 were chronic. − HBV infections: ID‐NAT results were categorised in four groups, and the findings showed that the levels of HBV DNA viral loads were different in the four different groups (*χ*2 = 275.02, *p* < 0.01). − HBV viral load distribution was significantly different between anti‐HBs positive and anti‐HBc positive samples (*χ*2 = 49.429, *p* < 0.01). Notably, only 42.03% donors were NAT repeated positive in the 138 repeat donors' follow up tests.	NAT screening of blood donations can reduce the risk of transfusion‐transmitted HBV infections. Positive proportions of anti‐HBs and anti‐HBc are correlated with the HBV viral load level. However, low level of viral load donors poses risks in HBV NAT assays, and show fluctuating state for HBV viral load and leads to non‐repeated NAT results during follow up studies.
Ali et al. [43] Pakistan	January 1, 2020 to November 30, 2022	HBV, HCV and HIV	59.708	Roche cobas 6800/8800 system using multiplex polym erase chain reaction kitmultiplex polymerase chain reaction kit cobas MPX test.	CLIA − immunoassay analyser (Architect i2000, Abbott diagnostics, Abbott park, IL) for HBsAg, anti‐HCV, HIV Ag‐Ab.	Serological methods: − HBsAg, 887 (1.5%) positive; − anti‐HCV 1015 (1.7%) positive; − HIV Ag‐Ab 47 (0.07%) positive. NAT method: 34 NAT‐reactive samples were identified − 31 cases of HBV; − 3 cases of HCV; TTIs (serology + NAT + ICT): − HCV 1.7% (*n* = 1018); − HBV 1.5% (*n* = 918); − HIV 0.07% (*n* = 47). Residual risks (RRs) of TTIs: − HBV ‐ 8.6 per million ‐ risk reduction ‐ 48%, 9%; − HCV 0.8 per million ‐ risk reduction ‐ 94%, 5%.	NAT implementation has improved blood safety. The parallel use of serology and NAT screening of donated blood would be beneficial.
Dettori et al. [36] Italy	January 2006 to August 2007	HBV	22.765	HBV‐DNA, HCV‐RNA, and HIV1‐RNA using the NAT generic procleix ultrio assay (chiron, emeryville, CA, USA). HBV‐DNA confirmation: Real time PCR (roche COBAS TaqMan 48 HBV test; roche molecular system, branchburg, NJ, USA; detection limit 6 IU/mL). HBV‐DNA sequencing and genotype determination.	HBsAg, Anti‐HBc, Anti‐HBe, HBeAg, Anti‐HBs, Anti‐HCV (AXSYM, Abbott laboratories, Abbott park, IL, USA). Anti‐HIV1/2 (vironostika HIV1/2 Ab/Ag, bioMerieux).	Serological methods: − HBsAg 12 (0.05%); − Anti‐HCV 14 (0.06%); − Anti‐HIV 3 (0.01%). NAT method: Generic NAT identified 31 (0.13%) reactive sera. − HBV‐DNA 15 (0.07%); − HCV‐RNA 8 (0.04%); − HIV1‐RNA 3 (0.01%); − Undetermined 5 (0.02%) ‐ these 5 samples were also HBsAg‐negative. *7 was positive to cobas TaqMan or the in‐house PCR after ultracentrifugation.	The data indicate that in areas with a low HBV endemicity, single NAT assays may not always identify blood donations with very low HBV‐DNA levels.
Dow et al. [37] Scotland	July 1999 to June 2003	HCV	1. 117. 681	HCV NAT minipool donation testing (in house). HCV genotyping: Restriction fragment length polymorphism (RFLP).	Anti‐HCV: − HCV immunoblots RIBA‐3 − Innolia HCVIII Update − HCV EIAs ortho HCV 3·0 with enhanced SAVe ‐ biorad monolisa HCV 3·0 plus version 2 *The HCV serotyping (HC02) EIA from Abbott/Murex was used on all anti‐HCV positive referrals. *Abbott PRISM HCV chemiluminescent immunoassay (ChLIA) ‐ routine donor anti‐HCV test. HCV antigen: Recently marketed Trak‐C second‐generation assay: − Ortho HCV antigen enzyme immunoassay; − Ortho Trak‐C EIA (second‐generation HCV antigen).	Serological methods: − anti‐HCV‐positive 110 (0·0098%) NAT method: − HCV NAT positive: 92 (83%); − HCV NAT negative: 18 (16%).	The individual HCV antigen testing should not be considered as equivalent to HCV NAT minipool screening. Trak‐C antigen testing may be considered as a suitable confirmatory assay for isolated HCV NAT reactivity.
Fang et al. [35] South Africa	1999	HCV, HIV‐1	19.709 ‐High HIV prevalence (HP): 10.632 ‐Low HIV prevalence (LP): 9.077	TMA ProcleixTM HIV‐1/HCV assay (chiron corporation, emeryville, CA).	HCV: − anti‐HCV reactivity − HBsAg HIV‐1: − HIV‐1 p24 antigen − anti‐HIV‐1/2 reactivity, *HIV seroconversion (STARHS) based on a sensitive/less‐sensitive enzyme immunoassay (‘detuned’ EIA). The vironostika HIV‐1 microelisa (bioMérieux Industry, raleigh, NC) assay for anti‐HIV was modified for ‘detuned’ EIA procleix assay using the SIA HCV RIBA 3·0 (chiron corporation).	− HIV was 45 times more prevalent in the HP (159 positives) than in the LP donor group)3 positives). All seven HIV‐1 p24 antigen‐positive samples in the study were also HIV NAT positive. Two HIV NAT‐positive samples were anti‐HIV negative. Assuming a 15‐day earlier detection by HIV NAT compared with antibody tests, these incidence rates project that NAT may intercept an additional 23 (95% confidence interval: 15–33) HIV‐positive donations per year. – HCV, two viral RNA and antibody‐positive samples (one from the LP group and one from the HP group) and no NAT yield cases were found in the study.	Implementation of routine NAT blood screening would allow elimination of HIV‐1 p24 antigen testing and improve the safety of the blood supply in South Africa.
Grabarczyk et al. [38] polland	2000 to 2016	HCV	17.502.739	Minipools of plasma (cobas ‐roche): TMA (procleix HCV/HIV‐1 − gen‐probe incorporated).	Anti‐HCV: HCV ELISA V3.0 (Ortho‐Clinical diagnostics, Inc a Johnson&Johnson company, raritan); Architect Anti‐HCV (ABBOTT, Wiesbaden, Germany); Vitros aHCV (Ortho‐Clinical diagnostics, Wycombe buckinghamshire, United Kingdom). Others: HCV RNA positive samples identified by NAT ‐ screening assays: – Chemiluminescence assays (CMIA) vitros aHCV (Ortho‐Clinical diagnostics); – Architect Anti‐HCV (ABBOTT); ‐electrochemiluminescence assay (ECLIA) —Elecsys Anti‐HCV II (roche diagnostics GmbH, mannheim, Germany); – Furth‐generation enzyme‐linked immunosorbent assay (ELISA) —monolisa HCV Ag‐Ab Ultra V2 (Bio‐Rad, Marnes‐la‐Coquette, France).	– HCV‐seronegative infections were identified in 126 donations. Frequency of NAT yields was decreasing over time. Of the initial 126 seronegative index cases 106 were retested: 34 (32.1%) were reactive in IV EIA, 12 (11.3%) in ECLIA, and 2 (1.9%) in CMIA. – The lowest viral load (VL) correlated with absent anti‐HCV and HCV Ag, while VL was highest when the antigen was detectable and then it decreased when anti‐HCV appeared at a level detectable by sensitive third generation tests while retesting. – The proportion of genotype 1 was 38.9% in samples positive only for HCV RNA and 71.4% in samples that were anti‐HCV reactive in re‐testing. In parallel, genotype 3 frequency was 50% in the former group and 21% in the latter.	The paper demonstrated high effectiveness of NAT in prevention of HCV transmission from seronegative donors. The highest clinical sensitivity, as assessed by NAT yields testing, was demonstrated by the IV generation tests, while among the third‐generation assays, ECLIA showed higher sensitivity than CMIA.
Houareau et al. [39] Germany	2006 to 2015	HBV	31.562.556	Pool‐NAT screening and ID‐NAT (LOD < 12 IU/mL).	Anti‐HBc and Anti‐HBs screening.	– Of all donations screened, 70.671 were anti‐HBc reactive but HBsAg negative (0.22%). – The proportion of repeat donors with these test results decreased significantly from 0.25% in 2007 to 0.08% in 2015. − In the entire study period, 82 HBV‐NAT‐positive donations were identified. Of these, 47 donations were only identified by ID‐NAT, of these, 5 were tested positive in ID− and pool‐NAT. − A total of 54.203 anti‐HBc‐reactive units were discarded either due to possible infectiousness (NAT positive or anti‐HBs concentration < 100 IU/L) or because no further testing was performed.	Anti‐HBc screening has improved blood safety in Germany. HBVNAT‐positive donations were identified after ID‐NAT was triggered by the initial reactive anti‐HBc result.
Madeira et al. [44] Brazil	January 2018 to December 2019	HBV, HCV and HIV	325.934	RT‐PCR to NAT HIV/HCV/HBV Biomanguinhos—FIOCRUZ kit.	Chemiluminescence methodology: Hepatitis B (HBsAg) Hepatitis C (anti‐HCV) HIV (anti‐HIV).	A total of 1.496 (0.46%) blood bags were reactive for HBV, HCV, or HIV in serological and/or NAT tests. – The highest prevalence of HBV occurred in the 8th RH, with a reagent serology of 0.34% and a positive NAT of 0.17%. − For HCV, the prevalence for reagent serology was 0.28%, while that for NAT was 0.02%, which occurred in the 20th RH. − For HIV and for NAT, the prevalence of blood donors with positive serology occurred in the 20th RH, at 0.25% and 0.04%, retrospectively. – The 13th RH had the highest prevalence of HIV in relation to NAT, that is, conventional serology in concomitance with NAT technology, at 0.07%. – During the 2‐year period, only 1 reactive donor in the 9th was found for NAT (HBV), in a diagnostic window.	This research showed a high donor rate in the diagnostic window period, considering the proportion of the population analysed. Such information is important to prove the need for NAT implementation, which is complementary to serology, improving transfusion safety.
Makroo et al. [42] India	June 2004 to January 2005	HBV, HCV and HIV‐1	12.224	Procleix ultrio assay (chiron corporation, emeryville, CA) using TMA.	HIV antibody, HCV antibody; and hepatitis B surface antigen (HBsAg): Conducted at each blood bank using different ELISA kits (20 different kits, full list disponible at the referenced article).	Of the 12.224 samples tested, 209 (1.71%) were seroreactive: − 133 samples (1.09%) were reactive by ultrio assay; − 84 samples were seroreactive but NAT non‐reactive. There were 8 NAT yield cases: 1 HIV, 1 HIV‐HCV co infection, and 6 HBV. The NAT yield for all three viruses was 1 in 1528 (0.065%).	The findings showed that NAT allowed rapid detection of three prevalent viruses that cause serious infections and its semi‐automated platform allowed individual testing in our low volume setting. It was estimated NAT could interdict 3272 infectious donations a year among our approximate 5 million annual donations.
Operskalski et al. [47] EUA	July 1974 to June 1980	HCV	5.387	RT‐PCR assay (cobas Amplicor HCV monitor, version 2.0, roche diagnostic Systems, Indianapolis, IN); TMA (gen‐probe Laboratories, San Diego, CA).	HCV 2.0 ELISA, ortho diagnostics, raritan; RIBA (RIBA HCV 2.0 SIA, chiron corporation, emeryville, CA, and ortho diagnostics, raritan, NJ).	– A total of 156 recipients of components from 180 anti‐HCV‐reactive donors were identified: − 107 of these were HCV‐naïve before transfusion and received a single, confirmed seropositive unit; − 94 (88%) became infected; − 85 recipients had donors whose HCV RNA level was quantifiable by RTPCR; − 83 (98%) seroconverted. − Of the remaining 22, a total of 10 received units positive for HCV RNA detected only by TMA; all 10 recipients seroconverted. − Of the remaining 12 recipients of anti‐HCV+, TMA‐negative units, 1 recipient seroconverted. − By the combined HCV RNA assays (RT‐PCR and TMA), 93 of 94 transmitting donations (99%) tested HCV RNA‐positive. In only 1 case was there transmission without HCV RNA detection.	High rates of transmission were seen at all levels of viraemia, and one donor transmitted with undetectable levels in the TMA assay. Current HCV RNA testing will therefore not interdict all infectious units, even with single‐donation testing, and serologic screening must be continued.
Altunay et al. [46] Turkey	July 2007 and October 2008	HBV: Anti‐HBc positive and Anti‐HBs negative	12.858	TMA with procleix nitro kit (Chiron‐ABD) confirmation with procleix ultrio kit.	Total serum: Automatic micro ELISA instrument (Biomerux‐France): − Anti‐HCV and HBsAg using vironostica − Nnotest HCV Ab III; − Hepa‐nostica Ultra HbsAg. 12,858 Hbs‐Ag negative cases were tested for: − Anti‐HBc and Anti‐HBs using Hepanostica HBc uniform total test kit; − Anti‐HBs kit (bio‐Kit‐Spain).	Serological methods: Total: 5.1% (658/12,858) of the total 12,858 HBs‐Ag negative samples were Anti‐HBC positive, HBS‐Ag negative and Anti‐HBs negative. − Anti‐HBc: 2748 positive; − Anti‐HBc‐10110 negative. 2748 Anti‐HBc positive samples: − 658 (24%) were Anti‐HBs negative; − 2090 (76%) were Anti‐HBc positive. NAT method: 12,858 HBs‐Ag negative plasma samples: − 9 (0007%) were detected positive (6 (0.91%) were HBV‐DNA positive); − 12,849 (99.93%) were detected negative.	Taking into account the 5% reduction of blood donors due to Anti‐HBc positivity in the blood banks, the presence of higher HBV‐DNA negativity (99.09%) in isolated anti‐HBc cases.

Abbreviations: ALT: alanine aminotransferase enzyme; CLIA: chemiluminescence immunoassay methodology; CMIA: chemiluminescence assays; ECLIA: electrochemiluminescence immunoassay; EIA: enzyme immunoassay; ELISA: enzyme‐Linked Immunosorbent Assay; HBV: hepatitis B virus; HBC: antibody against viral core antigen of HBV; HCV: hepatitis C virus; HIV: human immunodeficiency virus; HBsAg: hepatitis B virus surface antigen; HBeAg: hepatitis B virus E antigen; HBc: anti‐hepatitis B core antigen; HP: high prevalence; ID‐NAT: individual donation nucleic acid test; LOD: limit of detection; LP: low prevalence; MP‐NAT: mini‐pool nucleic acid test multiplex assay; MPX: multiplex; MEIA: microparticle enzyme immunoassays; OBI: occult hepatitis B infection; PCR: qualitative polymerase chain reaction; qPCR: real time quantitative polymerase chain reaction; RT‐PCR: reverse transcriptase–polymerase chain reaction; TMA: Transcription Mediated Amplification; TTIs: transfusion‐transmitted infections; VL: viral load; WP: window period.

Occult infection and diagnostic WP were not reported as cases in 10 studies (34.5%) [20, 26, 30, 36, 38, 39, 42, 46, 47, 48]. In 13 studies (68.4%), donors during the period of occult HBV infection (OBI) were identified [21, 22, 23, 25, 27, 28, 29, 31, 32, 33, 34, 40, 41] and 16 studies (55.2%) reported cases of WP [21, 23, 24, 25, 28, 29, 31, 32, 34, 35, 37, 40, 41, 43, 44, 45].

In nine studies that analysed only HBV infection [23, 27, 29, 30, 34, 36, 39, 45, 46], cases of occult infection were identified, and six also identified cases in the diagnostic WP [23, 29, 31, 34, 36, 45]. A specific article [45] identified only cases in the diagnostic WP. In 15 articles analysing all three infections, two identified cases of WP [24, 40], and nine (75%) found cases of OBI [21, 22, 25, 28, 32, 33, 34, 40, 41].

The seroconversion period identified for each infection, when comparing the studies of Li et al. [21] and Niazi et al. [25] showed similarity for HCV infection, ranging from 64 to 70 days. Regarding WP, the highest number of cases were for HBV infection, followed by HIV and HCV. In total, there were 154 cases of WP for HBV, ranging from 30 to 90 days; 48 for HIV, ranging from 10 to 22 days, and 34 for HCV ranging from 55 to 160 days. For occult HBV infection, a total of 832 cases were reported in the selected studies.

Among 19 of the 29 articles (65.5%), the residual risk (RR) of infection transmission was not estimated [22, 23, 26, 27, 28, 29, 30, 31, 32, 33, 34, 36, 37, 39, 42, 44, 46, 47, 48]. In this systematic review, the studies showed a total number of donors with positive serology and positive NAT of 4.444 for HBV, 319 for HCV, and 1239 for HIV. For the negative serology/positive NAT ratio, the quantitative was 3954 for HBV, 246 for HCV, and 54 for HIV (Table 2).

**TABLE 2:** Serological and molecular characteristics, residual risk, window period and cases of occult infection, according to HBV, HCV and HIV infection.

Study	Number of donors	Serology (+)/NAT (+)				Serology (−)/NAT (+)		Residual risk (RR)	Cases of window period (days)	Cases of occult infection
		HBV	HCV	HIV	HBV	HCV	HIV			
Kosan et al. [20]	18.200	297	9	3	11	2	0	HBV: 1:1.654 HCV: 1:9.100	NR	NR
Li et al. [21]	10.824	48	10	0	12	1	NR	HBV: 1:900 (0.11%) HCV: 1:10.000 (0.01%)	HBV: 0 HCV: 1 (64 days)	HBV: 12
Lin et al. [22]	5.973	6	1	—	8	—	—	NR	NR	HBV: 8
Louisirirotchanakul et al. [23]	175	0	—	—	175	—	—	NR	HBV: 15 (30–90 days)	HBV: 48
Moiz et al. [24]	41.304	NR	NR	NR	7	27	1	HBV: 1: 10.900 HCV: 1: 13.900 HIV: 1: 62.600	HBV: 7 (36.1 days) HCV: 27 (52.67 days) HIV: 1 (12.24 days)	NR
Niazi et al. [25]	54.438	NR	NR	NR	23	4	0	HBV: 62.5: 1.000.000 HCV: 4.4: 1.000.000	HBV: 3 (39.5 days) HCV: 4 (70 days) HIV: 0	HBV: 14 (61%) (6 non‐WP or OBI)
Selim et al. [26]	12.032	NR	NR	NR	0	5	2	NR	NR	NR
Yoshikawa et al. [27]	328	26	—	—	3	—	—	NR	NR	HBV: 3
Vermeulen et al. [40]	732.250	435	29	720	62	1	16	HBV: 48.45 (1: 20.642) HCV: 0.061 (1:16.226.411 HIV: 6.98 (1: 143.226)	HBV: 20 HCV: 1 HIV: 16	HBV: 36
Barbosa et al. [48]	50	—	—	12	—	—	0	NR	NR	—
Cable et al. [41]	649.745	371	15	241	21	0	0	HBV early WP: 1: 82.359 HBV 2^nd^ WP: 1.008.000 HIV repeat donors: 3.17: 1.000.000 HIV lapsed donors: 2.93: 1.000.000	HBV: 8 HCV: 0 HIV: 6	HBV: 13
Fiedler et al. [32]	46.151.554	NR	NR	NR	29	61	20	NR	HBV: 4 HIV: 1	HBV: 23
Kleinman et al. [45]	704.902	87	—	—	23	—	—	HBV: 1:370.588	HBV: 4 (7–22 days)	NR
Sgourou et al. [33]	38.264	52	8	0	7	0	0	NR	NR	HBV: 7
Stolz et al. [34]	306.000	24	10	5	7	NR	NR	NR	HBV: 2	HBV: 5
Wang et al. [28]	826.044	0	—	—	413	5	13	NR	HBV: 68	HBV: 221
Yang et al. [29]	65.800	6	—	—	59	—	—	NR	HBV: 3	HBV: 33
Zhang et al. [30]	11	—	27	—	—	9	—	—	NR	—
Wu et al. [31]	1.160.355	2.725	—	—	3.042	—	—	—	HBV: 19	HBV: 409
Ali et al. [43]	59.708	NR	NR	NR	31	3	0	HBV: 8.6 per million; HCV: 0.6 per million	Infectivity and NAT detectability: − HBV: 10.3 − HCV: 1.3 − HIV: 2.9 NAT and CLIA detectability: − HBV: 23.6 − HCV: 32.6 − HIV: 8.6	NR
Dettori et al. [36]	22.765	12	—	—	8	—	—	NR	NR	NR
Dow et al. [37]	1.117.681	—	92	—	—	1	—	NR	HCV: 1 (55–160 days)	NR
Fang et al. [35]	19.709	—	2	159	—	NR	NR	Antibody and p24 antigen: 0·34 (/10,000) NAT: 0·17 (/10,000); Projected yield of NAT:0·26 (/10,000)	HIV: 24 − Clade B Ab: 22 days Ag: 16 days NAT: 11 days − Clade C Ab: 25 days Ag: 20 days NAT: 10 days HCV: NR	NR
Grabarczyk et al. [38]	17.502.739	—	NR	—	—	126	—	0.3 to 1.2 per 1 million donations	HCV: 21	NR
Houareau et al. [39]	31.562.556	82	—	—	NR	—	—	NR	NR	NR
Madeira et al. [44]	325.934	178	10	88	1	0	0	NR	HBV: 1	NR
Makroo et al. [42]	12.224	101	13	11	6	1	2	NR	NR	NR
Operskalski et al. [47]	5.387	—	93	—	—	NR	—	NR	NR	NR
Altunay et al. [46]	12.858	NR	—	—	6	—	—	NR	NR	NR

Abbreviations: Ab: antibody; Ag: antigen; CLIA: chemiluminescence immunoassay methodology; HBV: hepatitis B virus; HBC: hepatitis C virus; HIV: human immunodeficiency virus; NAT: nucleic acid amplification testing; NR: not reported.

### 3.1 Risk of Bias and Applicability

**FIGURE 2:**
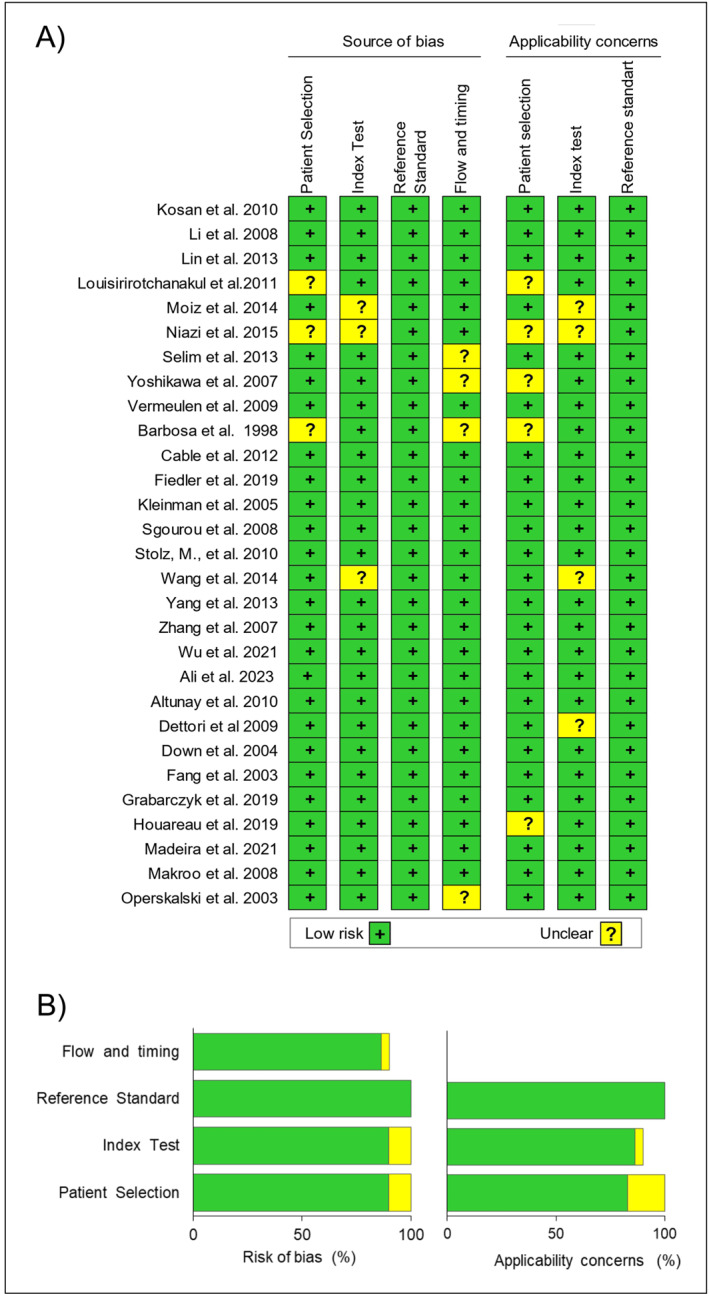
(A) Quality assessment of included works by the QUADAS‐2 tool. (B) Risk of bias and applicability concerns graph.

The methodological quality of the studies included in this systematic review is based on QUADAS‐2 (Figure 2). Most studies exhibited a low risk of bias (89.6%) concerning the domains of patient selection and index test [20, 21, 22, 26, 27, 29, 30, 31, 32, 33, 34, 35, 36, 37, 38, 39, 40, 41, 42, 43, 44, 45, 46, 47], while 82.8% demonstrated a low risk of bias for the flow and timing domain [20, 21, 22, 23, 24, 25, 28, 29, 30, 31, 32, 33, 34, 35, 36, 37, 38, 39, 40, 41, 42, 43, 44, 45, 46]. For the reference standard domain, there was a 100% low risk of bias [20, 21, 22, 23, 24, 25, 26, 27, 28, 29, 30, 31, 32, 33, 34, 35, 36, 37, 38, 39, 40, 41, 42, 43, 44, 45, 46, 47, 48]. Unclear information was noted in 27.5% of the publications [23, 24, 25, 27, 28, 33, 47, 48]. Applicability concerns with a low risk ranged from 82.8% to 86.0% for the patient selection and index text domains [20, 21, 22, 26, 29, 30, 31, 32, 33, 34, 36, 39, 40, 41, 45]. Unclear information for the patient selection and index domains were present in 17.2%–13.8% of studies [23, 24, 25, 27, 28, 36, 39, 48]. Conversely, the reference standard domain consistently exhibited a 100% low risk of bias [20, 21, 22, 23, 24, 25, 26, 27, 28, 29, 30, 31, 32, 33, 34, 35, 36, 37, 38, 39, 40, 41, 42, 43, 44, 45, 46, 47, 48].

### 3.2 Meta‐Analysis and Publication Bias

#### 3.2.1 HBV, HBC, and HIV Serological Negative Tests and NAT Positive Results

The meta‐analysis of HBV serological negative tests and NAT positive results included 17 studies involving 49,578,146 HBV seronegative donors, with 851 testing positive for HBV NAT [20, 21, 22, 23, 27, 28, 29, 32, 33, 34, 36, 40, 41, 42, 44, 45, 46]. Regarding serological tests negative for HCV and positive for NAT, 10 studies were selected, with six studies analysing 47,751,096 seronegative donors and reporting 71 HCV NAT positive results [20, 21, 28, 29, 40, 42]. As for negative serological tests for HIV and positive NAT, seven studies were selected, with four studies analysing 47,722,072 seronegative donors and reporting 51 NAT positive results (Table 3) [25, 29, 37, 39].

**TABLE 3:** Distribution of event rates for HBV, HCV and HIV across the different tests applied.

	Serological negative tests and NAT positive results	Positive NAT results recovered after negative serology	Serological tests and NAT positive results
HBV	1:58,259	1:2545	1:19,797
HCV	1:672,550	1:4289	1:10,056
HIV	1:935,726	1:17,778	1:1419

*Note:* The rates refer to the ratio between the number of donations tested and the number of positive cases for HCV: hepatitis C virus; HBV: hepatitis B virus; and HIV: human immunodeficiency virus.

**FIGURE 3:**
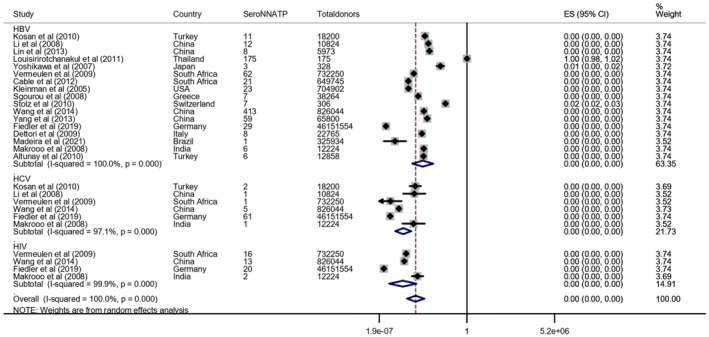
Forest plot of the Meta‐analysis of the frequency of concordance of negative serological tests and NAT positive for HBV, HBV and NAT concerning the total number of donors according to random effects analysis. SeroNNATP (serology negative and positive NAT), CI = confidence interval, weight (%), ES = effect size.

The pooled frequency was 0.00 (0.00–0.00) for HBV, HCV, and HIV, with high heterogeneity across all three (I^2^ = 100.0%, *p* < 0.001) (Figure 3). The Begg's test for HBV and HCV revealed significant publication bias (*p* < 0.01), and showed no publication bias for HIV (*p* = 0.071). The Egger's and Begg's tests found significant publication bias only for HCV (*p* = 0.017, *p* < 0.001), and showed no publication bias for HBV and HIV (*p* = 0.191, *p* = 0.217) (Supporting Information S4: File 4).

#### 3.2.2 Positive NAT Results Recovered After Negative Serology

NAT results were recovered on all HBV negative serological tests (155,250 donors), with a positive NAT result for 61 donors in three studies [24, 25, 43]. In four studies, the NAT results were recovered on all HCV negative serological tests (167,282 donors), with a positive NAT result for 39 donors [24, 25, 26, 43]. For negative HIV serological tests (53,336 donors) [21, 23], three positive NAT results were retrieved in two articles (Table 3) [24, 26].

**FIGURE 4:**
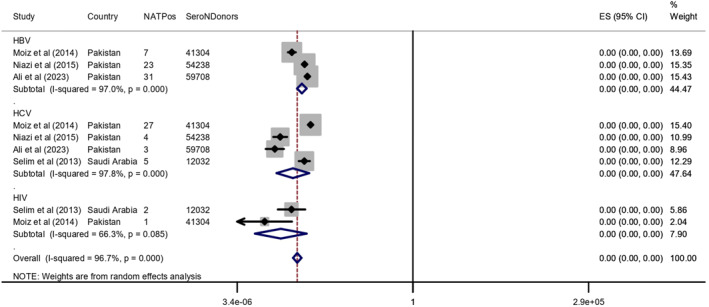
Forest plot of the Meta‐analysis of the frequency of concordance of NAT positive for HBV, HCV and HIV tests recovered after negative serology concerning the total number of donors. NATPos (positive NAT), SeroNDonors (negative donors serological tests), CI = confidence interval, weight (%), ES = effect size.

The pooled frequency was 0.00 (0.00–0.00) for HBV, HCV and HIV, with high heterogeneity for HBV (I^2^ = 97.0%, *p* < 0.001) and HCV (I^2^ = 97.8%, *p* < 0.001) and lower for HIV (I^2^ = 66.3%, *p* = 0.085) (Figure 4). Begg's and Egger's tests showed significant publication bias for HBV and HCV (*p* < 0.01; *p* < 0.001). Begg's test did not show significant publication bias for HIV (*p* = 0.092) (Supporting Information S5: File 5).

#### 3.2.3 HBV, HBC, and HIV Serological Tests and NAT Positive Results

**FIGURE 5:**
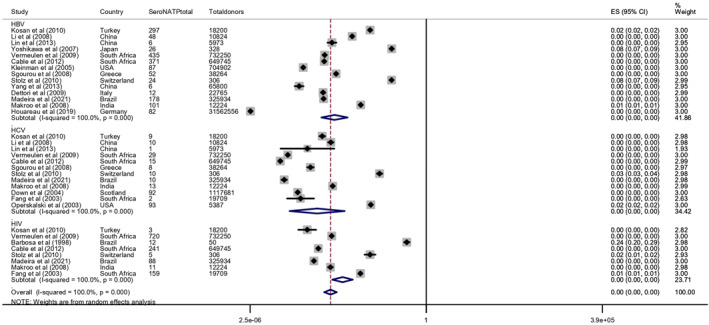
Forest plot of the frequency of concordance of positive serological tests and NAT for HBV, HCV, AND HIV concerning the total number of donors according to random effects analysis. SeroNATPtotal (serology and NAT positive). CI = confidence interval, weight (%), ES = effect size.

Fourteen studies analysed 34,150,071 positive HBV NAT and serological tests (donors, yielding 1725 HBV serum/NAT positives [20, 21, 22, 27, 29, 33, 34, 36, 39, 40, 41, 42, 44, 45] 12 studies analysed positive HCV NAT and serological tests involving 2,936,497 donors, with 292 HCV serum/NAT positives [20, 21, 22, 33, 34, 35, 37, 40, 41, 42, 44, 47]. Finally, eight studies involved positive HIV NAT and serological tests from 1,758,418 donors, with 1239 HCV serum/NAT positives (Table 3) [20, 34, 35, 40, 41, 42, 44, 48]. The pooled frequency was 0.00 (0.00–0.00) for HBV, HCV, and HIV (Figure 5), with high heterogeneity across all three (I^2^ = 100.0%, *p* < 0.001). Begg's and Egger's tests showed no significant publication bias for HBV, HCV, and HIV (*p* = 0.283, *p* = 0.839; *p* = 0.220, *p* = 02.70; *p* = 0.372, *p* = 0.263, respectively) (Supporting Information S1: File 6).

## 4 Discussion

According to the World Health Organisation, approximately 118,54 million blood donations are collected worldwide. Of these, 40% are sourced from high‐income countries, where 16% of the world's population resides [49]. It is worth highlighting there is a marked difference in the level of access to blood between low‐ and high‐income countries, and one of the biggest challenges in transfusion services is to provide safe blood products that meet patients' needs. In many developing countries, the risk of transfusion‐transmitted infections remains a fact, and prevention of these infections is a concern; measures such as reducing unnecessary transfusions, using biological materials only from regularly screened volunteer donors, excluding donors with risk factors, and screening all donated blood are not always implemented consistently [50].

Within this global context, the immunological WP remains a critical vulnerability in transfusion safety, particularly for hepatitis B virus (HBV). Data from the diagnostic WP showed the highest number of HBV infections, with 154 cases in 82,778,498 donors. This finding is consistent with previous large‐scale studies showing that HBV accounts for the majority of WP detections, reflecting its complex virological and immunological characteristics [10, 11].

Vermeulen et al. [9] demonstrated that, over 10 years of individual donation NAT screening in South Africa, approximately 7.7 million donations were tested, and a large number of HIV WP donations were intercepted, with only one confirmed case of transfusion‐transmitted HIV. These observations support the effectiveness of NAT in substantially reducing HIV transmission risk during the WP, even in high‐prevalence settings. Using NAT, Scuracchio et al. [51] identified two donors in the WP after screening 47,866 donations over 18 months. Fiedler et al. [32] when checking the effectiveness of NAT for HIV, HBV, and HCV between 2008 and 2015 in Germany, obtained an average of three to six donations per year with HBV infections detected by NAT screening alone, resulting in 29 positive cases, 20 HIV and 61 HCV cases were detected.

Taken together, these findings indicate that although WP infections are relatively uncommon, their interception remains clinically meaningful, as even a small number of infectious donations can result in preventable transmission events. Accordingly, an important area for future investigation is the identification and prediction of populations at increased risk for WP events, defined as recent infections with still negative serological markers. This consideration is particularly relevant for HIV, HBV, and HCV, since the risk of WP infections is concentrated in groups characterised by recent exposure and/or higher background prevalence. Evidence from large donor cohorts consistently shows that WP detections occur more frequently among first‐time donors and in regions with elevated viral prevalence [9, 11, 52]. In addition, donors reporting high‐risk behaviours, including injection drug use, sexual exposure among men who have sex with men, transgender and gender‐diverse individuals, and engagement in sex work, require attention [53, 54]. Collectively, these observations underscore the potential value of risk‐stratified surveillance approaches, which may support more targeted screening strategies and inform refinements in donor selection and screening policies, particularly in settings with a high prevalence of transfusion‐transmissible infections.

Among donors who presented either positive or negative serology and positive NAT, HBV infection was predominant. Despite HBV being an immunopreventable disease, attributable to vaccines, hepatitis B continues to be one of the most significant public health problems. Moreover, the infection may be associated with the cumulative effect of behavioural risk factors, such as unprotected sex, use of injectable illicit drugs, and exposure to blood and blood products [55].

Regarding serological screening, the sensitivity of hepatitis B virus surface antigen (HBsAg) assays has improved considerably over time but remains limited to detecting seroconversion during the WP or in samples with very low viral load after decades of chronic infection or clinical recovery [56]. As highlighted by Lelie et al. [11] these limitations are particularly evident among repeat donors, in whom serological markers may remain undetectable despite ongoing low‐level viraemia. The development of NAT has made it possible to identify these cases, enhancing the safety of diagnostic testing.

According to the study by Mulrooney‐Cousin and Michalak, [57] occult HBV infections are increasingly recognized as a problem, especially in regions with high HBV prevalence. In our systematic review, we identified 832 cases of occult hepatitis B infections (OBI). Patients with OBI are HBsAg‐negative, with hepatitis B virus DNA detectable through molecular biology, either in the presence or absence of antibodies to the HBV core antigen (anti‐HBc), with or without antibodies to HBsAg (anti‐HBs). [58] This condition often arises as a consequence of acute hepatitis resolution and can continue indefinitely after HbsAg clearance [57]. However, El Chaar et al. [59] reported several conditions responsible for OBI infections, including recovery from past infection, as defined by the presence of anti‐HBs, chronic hepatitis with escape mutant surface genes, chronic carriers without any marker of HBV infection other than HBV DNA (referred to as seronegative), and chronic carriers with very low HBsAg levels detectable only by the presence of anti‐HBc alone.

An analysis of 2.6 million Australian donors tested for HBV‐NAT, anti‐HBc and HBsAg, between 2010 and 2012 showed a substantially higher prevalence of OBI compared to acute HBV infections during the serological WP, emphasising the importance of anti‐HBc in HBV donor screening [57]. The infectivity of blood from donors with OBI remains unclear; however, it depends on the infectious dose and the immunocompetence of the recipient [55].

Kleinman et al. [60] consider residual risk to be an important statistical tool used in hemotherapy to analyse transfusion safety, expressed as the likelihood of contracting a particular infection through transfused blood. As discussed by Lelie et al. [12] residual risk estimates are highly dependent on model assumptions, assay sensitivity, and donor population characteristics, limiting direct comparisons across studies. Consequently, it was not possible to quantitatively compare residual risk estimates among the studies included in this review, as populations, methodologies, and reporting practices varied considerably. Nevertheless, available evidence indicates that the prevalence of serological marker positivity may vary across regions, reflecting differences in donor population characteristics, including the proportion of first‐time versus repeat donors, prior screening policies, and the specific serological assays employed, rather than uniform differences in assay performance [9, 11, 52]. These contextual factors are critical when interpreting NAT yield and residual risk estimates across different settings.

The findings of this systematic review support the role of NAT as an important component of blood donor screening strategies when used in conjunction with serological testing. Evidence from national and international studies indicates that NAT improves the detection of HIV, HCV, and HBV infections, particularly those missed during the serological WP, thereby contributing to a high standard of blood safety [7].

Cable et al. [41] also stated that the introduction of NAT has enhanced the safety of blood products by reducing the potential for HBV transmissions. Ali et al. [43] further suggested that the parallel use of serology and NAT for blood donor screening is beneficial, as NAT reduced the residual risk for HBV by 48.9% and for HCV by 94.5% in their prospective cross‐sectional study conducted between January 2020 and November 2022, which included 59,708 donors. The implementation of NAT technology associated with serological tests, as summarised in this systematic review, can contribute to evidence‐based practices. Grouped evidence is an increasingly important resource in the health sector. Systematic reviews provide valuable information not only to researchers and health care professionals but also to the susceptible society, and this is fundamental as a stimulus/trigger to study the dynamics of these infections.

The recovery and concordance of positive NAT from both negative and positive serological tests for HBV, HCV, and HIV confirm the reliability and safety of molecular tests. It is important to highlight that although the pooled measures in the meta‐analyses remained close to zero, the point estimates indicated significant agreement. The large denominator values in the analysis contributed to approximating the grouped measures to zero or close to zero; however, the evidence is clearly depicted in the forest plot figures of various meta‐analyses.

Given the limitations of the results in some studies included in this systematic review, it is important to report as much information as possible in a clear and robust manner to accurately address the objectives and results, while supporting the development of future studies, systematic reviews, and meta‐analysis. It is also noteworthy that most studies in the systematic review were methodologically different in terms of serology, meaning they did not adhere to a standardized approach. Variations in serological techniques, equipment brands, and kits used for the same infection limited the ability to compare techniques in terms of sensitivity. Improved harmonisation of screening methodologies would enhance comparability across studies and strengthen future systematic reviews and meta‐analyses.

### 4.1 Strengths and Limitations of the Study

To develop this systematic review and meta‐analyses, several publications of interest were examined across the five databases mentioned. The researchers, organised into working groups, held several meetings to define the most appropriate MeSH terms and descriptors, respecting the characteristics of each database. The eligibility criteria determined through consensus and the PICO (patient/population, intervention, comparison, and outcomes) strategy provided greater clarity and robustness to the research. The quality of the articles included in this systematic review, analysed according to the QUADAS‐2 tool, showed that most publications had a low risk of bias. The first limitation highlights that only publications in English were selected, which may have reduced the number of articles. The second limitation was the small number of publications included in the meta‐analyses to calculate the proportional pooled frequency of positive NAT retrieved after negative donor serology. The third limitation concerns the high heterogeneity of publications; however, most meta‐analyses did not show publication bias using Begg's and Egger's tests. The fourth limitation concerns the cost‐effectiveness involved in NAT implementation. As the objective of our systematic review is to investigate the safety of NAT technology in conjunction with serological tests for screening blood donors with HBV, HCV, and HIV, studies that described only cost‐effectiveness were excluded during the initial screening, as mentioned in the inclusion criteria; therefore, this topic could not be discussed in detail with the information aggregated in this review. However, of the 29 selected studies, 15 (51.7%) reported on the cost‐benefit of NAT, and only one study discussed the cost‐benefit of the NAT by describing the validation studies performed, as well as the sensitivity and the cost‐effectiveness of the established procedure [24].

Although the number of positive NAT results was small, this systematic review and meta‐analyses generate important evidence of the positive relationship between the improved safety of molecular technology and serological tests in screening blood donors for HBV, HCV, and HIV.

## 5 Conclusion and Future Direction

This systematic review and meta‐analysis evidenced the positive relationship and safety of molecular technology and serological tests in the screening process of blood donors with HBV, HCV, and HIV.

Despite the rigorous screening in blood banks, information from laboratories and clinical studies conducted in the general population may provide important knowledge regarding asymptomatic individuals, occult infections, and the diagnostic WP that impact the hemotherapy system. Other studies, including meta‐analysis and statistical techniques focused on identifying heterogeneity in the results, are important as they contribute to the establishment of adequate prevention, control strategies, and surveillance in blood banks.

## Author Contributions

J.J.V.T., L.D.de.P., and H.S.M. designed the study protocol and fully supported the research team. H.S.M., M.E.da.S.A., M.V.G.R., R.G.A., F.A.J., L.D.G.L., and D.de.C.M. organised the keyword blocks by consensus and searched the platform for data extraction and structuring into tables and figures. They also contributed to the introduction and methodological quality. J.J.V.T. and M.V.C.L. analysed the quality, accuracy, and validation of the keyword blocks as experts. M.V.C.L., D.A.B., L.D.de.P., C.M.da.S., D.S.S.L.L.‐N., and A.C.F.H.R.‐M served as judges to validate the extracted data for the tables and figures after reading the publications in PDF format, and to develop the meta‐analysis and structure the results. J.J.V.T., M.V.C.L., D.S.S.L.L.‐N. and A.C.F.H.R.‐M. assessed methodological quality using a publication bias checklist, validated the results, and performed statistical analyses. J.J.V.T., H.S.M., F.A.J., and A.C.F.H.R.‐M. contributed to the discussion, critical review, and synthesis of the data. All authors read and approved the manuscript in its entirety and agreed to its submission for publication after extensive critical review.

## Funding

The authors have nothing to report.

## Ethics Statement

The authors have nothing to report.

## Consent

All the authors have reviewed and approved the manuscript for publication.

## Conflicts of Interest

The authors declare no conflicts of interest.

## Permission to Reproduce Material From Other Sources

The authors have nothing to report.

## Supporting information


Supporting Information S1

Supporting Information S2

Supporting Information S3

Supporting Information S4

Supporting Information S5

Supporting Information S6

## Data Availability

The data that supports the findings of this study are available in the supplementary material of this article.
