# Supplementary material for: Improved Safety of Nucleic Acid Amplification Technology Combined With Serological Tests for Screening Blood Donors: A Systematic Review and Meta‐Analysis
Source: Rev Med Virol. 2026 Feb 21;36(2):e70117. doi: 10.1002/rmv.70117 (PMC12924692; doi:10.1002/rmv.70117)
Supplement: Supplementary file 2 — Supporting Information S2 [file RMV-36-e70117-s004.docx]

**S2. Supplementary file 2: MeSh terms used in the systematic review and meta-analysis**

| **PubMed Database**  **Strategy 1: search for MeSh terms** | **Embase Database** | **Web of science Database**  **Strategy Ti (title)** | **Scopus Database** | Cochrane library |
| --- | --- | --- | --- | --- |
| **Stage I - MeSh terms** | **Stage I- Search the free terms using three strategies:** Map to preferred term in Emtree; Limit to terms indexed in article as 'major focus'; Explode using narrower Emtree terms | **Stage I- Search the free terms using the strategy: Ti (title)** | **Stage I- Search for terms using the strategy: title, keywords and abstract** | **General strategy (title, abstract, Keywords)** |
| Block I *– “*nucleic acid amplification techniques” [Mesh] AND serologic tests" [Mesh] | Block 1 *-*'nucleic acid amplification techniques'/exp/mj **OR** 'nucleic acid amplification test'/exp/mj  **AND** 'serology'/exp/mj | Block 1 *-* Ti=(**Nucleic Acid Amplification Techniques) OR Ti=(N**ucleic Acid Amplification Test) OR Ti=(Nucleic Acid Amplification Technology) **AND Ti= (Serologic Tests)** | Block 1 *-* Ti=(**Nucleic Acid Amplification Techniques) OR Ti=(N**ucleic Acid Amplification Test) OR Ti=(Nucleic Acid Amplification Technology) **AND Ti= (Serologic Tests)** | ((**Nucleic Acid Amplification Techniques OR N**ucleic Acid Amplification Test OR Nucleic Acid Amplification Technology **AND Serologic Tests) AND (Blood Donors OR Blood donation) AND ( Hepatitis C OR Hepatitis B virus OR Hepacivirus OR Hepatitis B OR HIV))** |
| **Stage II- MeSh terms** | **Stage II- Ti (Title)** | **Stage II- Ti (Title)** | **Stage II- Ti (Title)** |  |
| Block 2: “blood donors” [Mesh] OR “blood donation” [Mesh] | Block 2 *-* 'blood donor'/exp/mj**)** | Block 2 *-* **Ti=(Blood Donors) OR Ti=(Blood donation)** | Block 2 *-* **Ti=(Blood Donors) OR Ti=(Blood donation)** |  |
| **Stage III - Mesh terms** | **Stage III- Ti (Title)** | **Stage III- Ti (Title)** | **Stage III- Ti (Title)** |  |
| Block 3: “hepatitis C" [Mesh] OR "hepatitis B virus" OR "hepacivirus" OR "hepatitis B" OR "HIV." | Block 3 *–* 'hepatitis c'/exp/mj OR 'hepatitis b virus'/exp/mj OR 'hepacivirus'/exp/mj OR 'hepatitis b'/exp/mj OR 'human immunodeficiency virus'/exp/mj | Block 3 *-* **Ti=( Hepatitis C) OR Ti=(Hepatitis B virus) OR Ti (Hepacivirus) OR Ti=(Hepatitis B) OR Ti=(HIV)** | Block 3 *-* **Ti=( Hepatitis C) OR Ti=(Hepatitis B virus) OR Ti (Hepacivirus) OR Ti=(Hepatitis B) OR Ti=(HIV)** |  |
| **Stage IV - Crossing with the boolean AND between the three blocks** | **Stage IV - Crossing with the boolean AND between the three blocks** | **Stage IV - Crossing with the boolean AND between the three blocks** | **Stage IV - Crossing with the boolean AND between the three blocks** |  |
| *“*nucleic acid amplification techniques” [Mesh] AND serologic tests" [Mesh] AND “blood donors” [Mesh] OR “blood donation” [Mesh] AND Block 3: “hepatitis C" [Mesh] OR "hepatitis B virus"[Mesh] OR "hepacivirus"[Mesh] OR "hepatitis B" OR "HIV"[Mesh] | Block 1 AND Block 2 AND Block 3 | Block 1 AND Block 2 AND Block 3 | Block 1 AND Block 2 AND Block 3 |  |
| **Strategy 2: Free term search** |  |  |  |  |
| We perform numerous searches by crossing free terms: tiab (title/abstract)  Block 1: **Nucleic Acid Amplification Techniques [**tiab] **N**ucleic Acid Amplification Test [tiab] Nucleic Acid Amplification Technology [**tiab] Serologic Tests** [**tiab]**  **Block 2: Blood Donation** [**tiab]** Blood Donation [**tiab]**  **Block 3: Hepatitis C** [**tiab] Hepatitis B Virus** [**tiab] Hepacivirus** [**tiab] Hepatitis B** [**tiab] HIV** [**tiab]** |  |  |  |  |
