# Supplementary material for: Improved Safety of Nucleic Acid Amplification Technology Combined With Serological Tests for Screening Blood Donors: A Systematic Review and Meta‐Analysis
Source: Rev Med Virol. 2026 Feb 21;36(2):e70117. doi: 10.1002/rmv.70117 (PMC12924692; doi:10.1002/rmv.70117)
Supplement: Supplementary file 3 — Supporting Information S3 [file RMV-36-e70117-s006.docx]

**S3. Supplementary file 3**

Studies selected and included in the systematic review and meta-analysis, according to the manuscript.

| 20. | Kosan E, Kocazeybek B, Altunay H, et al. Can the nucleic acid amplification test (NAT) be an alternative to the serologic tests? A prospective study, the results of 18,200 blood donors from the Turkish Red Crescent. *Transfus Apher Sci.* 2010;43(3):269-272. |
| --- | --- |
| 21. | Li L, Chen PJ, Chen MH, Chak KF, Lin KS, Tsai SJ. A pilot study for screening blood donors in Taiwan by nucleic acid amplification technology: detecting occult hepatitis B virus infections and closing the serologic window period for hepatitis C virus. *Transfusion.* 2008;48(6):1198-1206. |
| 22. | Lin KT, Chang CL, Tsai MH, Lin KS, Saldanha J, Hung CM. Detection and identification of occult HBV in blood donors in Taiwan using a commercial, multiplex, multi-dye nucleic acid amplification technology screening test. *Vox Sang.* 2014;106(2):103-110. |
| 23. | Louisirirotchanakul S, Oota S, Khuponsarb K, et al. Occult hepatitis B virus infection in Thai blood donors. *Transfusion.* 2011;51(7):1532-1540. |
| 24. | Moiz B, Moatter T, Shaikh U, et al. Estimating window period blood donations for human immunodeficiency virus Type 1, hepatitis C virus, and hepatitis B virus by nucleic acid amplification testing in Southern Pakistan. *Transfusion.* 2014;54(6):1652-1659. |
| 25. | Niazi SK, Bhatti FA, Salamat N, Ghani E, Tayyab M. Impact of nucleic acid amplification test on screening of blood donors in Northern Pakistan. *Transfusion.* 2015;55(7):1803-1811. |
| 26. | Selim HM, ElBashaar MA, ElWakil SG. Donor minipool NAT screening for HBV, HCV, and HIV: a 2-year experience in a private hospital in Saudi Arabia. *Comparative Clinical Pathology.* 2014;23:1125-1132. |
| 27. | Yoshikawa A, Gotanda Y, Minegishi K, et al. Lengths of hepatitis B viremia and antigenemia in blood donors: Preliminary evidence of occult (hepatitis B surface antigen-negative) infection in the acute stage. *Transfusion.* 2007;47(7):1162-1171. |
| 28. | Wang LN, Chang L, Xie YZ, et al. What is the meaning of a nonresolved viral nucleic acid test-reactive minipool? *Transfusion.* 2015;55(2):395-404. |
| 29. | Yang ZS, Xu L, Liu L, et al. Routine screening of blood donations at Qingdao central blood bank, China, for hepatitis B virus (HBV) DNA with a real-time, multiplex nucleic acid test for HBV, hepatitis C virus, and human immunodeficiency virus Types 1 and 2. *Transfusion.* 2013;53(10):2538-2544. |
| 30. | Zhang HQ, Li SB, Wang GH, Chen K, Song XG, Feng XY. Detection of hepatitis C virus core antigen for early diagnosis of hepatitis C virus infection in plasma donor in China. *World J Gastroenterol.* 2007;13(19):2738-2742. |
| 31. | Wu D, Wang X, Feng F, et al. Characteristic of HBV nucleic acid amplification testing yields from blood donors in China. *BMC Infect Dis.* 2021;21(1):714. |
| 32. | Fiedler SA, Oberle D, Chudy M, et al. Effectiveness of blood donor screening by HIV, HCV, HBV-NAT assays, as well as HBsAg and anti-HBc immunoassays in Germany (2008-2015). *Vox Sang.* 2019;114(5):443-450. |
| 33. | Sgourou A, Karakantza M, Theodori E, et al. Procleix Ultrio transcription-mediated amplification vs. serological blood screening in south-western Greece. *Transfus Med.* 2008;18(2):104-111. |
| 34. | Stolz M, Tinguely C, Graziani M, et al. Efficacy of individual nucleic acid amplification testing in reducing the risk of transfusion-transmitted hepatitis B virus infection in Switzerland, a low-endemic region. *Transfusion.* 2010;50(12):2695-2706. |
| 35. | Fang CT, Field SP, Busch MP, Heyns Adu P. Human immunodeficiency virus-1 and hepatitis C virus RNA among South African blood donors: estimation of residual transfusion risk and yield of nucleic acid testing. *Vox Sang.* 2003;85(1):9-19. |
| 36. | Dettori S, Candido A, Kondili LA, et al. Identification of low HBV-DNA levels by nucleic acid amplification test (NAT) in blood donors. *Journal of Infection.* 2009;59(2):128-133. |
| 37. | Dow BC, Munro H, Buchanan I, et al. Acute hepatitis C virus seroconversion in a Scottish blood donor: HCV antigen is not comparable with HCV nucleic acid amplification technology screening. *Vox Sang.* 2004;86(1):15-20. |
| 38. | Grabarczyk P, Kubicka-Russel D, Kopacz A, et al. Seronegative hepatitis C virus infection in Polish blood donors-Virological characteristics of index donations and follow-up observations. *J Med Virol.* 2020;92(3):339-347. |
| 39. | Houareau C, Offergeld R. Anti-HBc screening - is it worth the effort? Results of a 10-year surveillance programme covering more than 30 million donations in Germany. *Vox Sang.* 2019;114(5):459-466. |
| 40. | Vermeulen M, Lelie N, Sykes W, et al. Impact of individual-donation nucleic acid testing on risk of human immunodeficiency virus, hepatitis B virus, and hepatitis C virus transmission by blood transfusion in South Africa. *Transfusion.* 2009;49(6):1115-1125. |
| 41. | Cable R, Lelie N, Bird A. Reduction of the risk of transfusion-transmitted viral infection by nucleic acid amplification testing in the Western Cape of South Africa: a 5-year review. *Vox Sang.* 2013;104(2):93-99. |
| 45. | Kleinman SH, Strong DM, Tegtmeier GG, et al. Hepatitis B virus (HBV) DNA screening of blood donations in minipools with the COBAS AmpliScreen HBV test. *Transfusion.* 2005;45(8):1247-1257. |
| 46. | Altunay H, Kosan E, Birinci I, et al. Are isolated anti-HBc blood donors in high risk group? The detection of HBV DNA in isolated anti-HBc cases with nucleic acid amplification test (NAT) based on transcription-mediated amplification (TMA) and HBV discrimination. *Transfus Apher Sci.* 2010;43(3):265-268. |
| 47. | Operskalski EA, Mosley JW, Tobler LH, et al. HCV viral load in anti-HCV-reactive donors and infectivity for their recipients. *Transfusion.* 2003;43(10):1433-1441. |
| 48. | Barbosa EF, Carneiro-Proietti AB, Oliveira DR, Lima-Martins MV, Kroon EG, Ferreira PC. HIV-1 detection and subtyping by PCR and heteroduplex mobility assay in blood donors: can these tests help to elucidate conflicting serological results? *Transfus Sci.* 1998;19(1):39-43. |
| 42. | Makroo RN, Choudhury N, Jagannathan L, et al. Multicenter evaluation of individual donor nucleic acid testing (NAT) for simultaneous detection of human immunodeficiency virus -1 & hepatitis B & C viruses in Indian blood donors. *Indian J Med Res.* 2008;127(2):140-147. |
| 43. | Ali SM, Raza N, Irfan M, Mohammad MF, Kazmi FH, Fatima Z. Effectiveness of Using Nucleic Acid Amplification Test to Screen Blood Donors for Hepatitis B, Hepatitis C, and HIV: A Tertiary Care Hospital Experience From Pakistan. *Cureus.* 2023;15(1):e34216. |
| 44. | Madeira HS, da Silva CM, Scapini NC, de Peder LD, Teixeira JJV. Correlation between serology and nucleic acid amplification test in blood donors who are reactive for hepatitis B virus, hepatitis C and human immunodeficiency virus and evaluation of the epidemiological profile of infected people in blood centers in the State of Paraná. *Saudi Pharm J.* 2021;29(6):586-596. |

**Studies excluded in the systematic review, after reading the PDF file for not meeting the research objective.**

| 1. | Laperche S. Detection of the nucleic acids of hepatitis B and C viruses and human immunodeficiency virus for the biological screening of blood donations. Viral Hepatitis and Retrovirus Working Groups and Subgroup for Molecular Biology Applied to Transfusion Virology of the French Blood Transfusion Society. *Transfusion clinique et biologique : journal de la Société française de transfusion sanguine.* 1998;5(2):139-146. |
| --- | --- |
| 2. | Krenz-Weinreich A, Dennin RH. The status of hepatitis B virus-(HBV)-DNA in sera with various constellations of HBV specific markers: Application of different methods with respect to clinical requirements. *Klinisches Labor.* 1995;41(10):749-752. |
| 3. | Bruhn R, Lelie N, Busch M, Kleinman S, Int NATSG. Relative efficacy of nucleic acid amplification testing and serologic screening in preventing hepatitis C virus transmission risk in seven international regions. *Transfusion.* 2015;55(6):1195-1205. |
| 4. | Bujandric N, Grujic J, Obradovic ZB. Assessing donor suitability for blood donation: Utility of Geenius HIV 1/2 confirmatory assay. *Transfusion and Apheresis Science.* 2021;60(1). |
| 5. | Cappy P, Barlet V, Lucas Q, et al. Transfusion of HIV-infected blood products despite highly sensitive nucleic acid testing. *Transfusion.* 2019;59(6):2046-2053. |
| 6. | Coen S, Angeletti C, Piselli P, et al. A statistical model based on serological parameters for predicting occult HBV infection: implications for organ/blood donations. *New Microbiologica.* 2015;38(1):39-49. |
| 7. | Corrêa ASM, Lamarão LM, Vieira PCM, et al. Prevalence, incidence and residual risk of transfusion-transmitted HBV infection before and after the implementation of HBV-NAT in northern Brazil. *PLoS One.* 2018;13(12):e0208414. |
| 8. | Dodd RY, Notari EPt, Stramer SL. Current prevalence and incidence of infectious disease markers and estimated window-period risk in the American Red Cross blood donor population. *Transfusion.* 2002;42(8):975-979. |
| 9. | Busch MP, Glynn SA, Stramer SL, et al. A new strategy for estimating risks of transfusion-transmitted viral infections based on rates of detection of recently infected donors. *Transfusion.* 2005;45(2):254-264. |
| 10. | Grabarczyk P, van Drimmelen H, Kopacz A, et al. Head-to-head comparison of two transcription-mediated amplification assay versions for detection of hepatitis B virus, hepatitis C virus, and human immunodeficiency virus Type 1 in blood donors. *Transfusion.* 2013;53(10 Pt 2):2512-2524. |
| 11. | Hyland CA, Seed CR, Kiely P, Parker S, Cowley N, Bolton W. Follow-up of six blood donors highlights the complementary role and limitations of hepatitis C virus antibody and nucleic acid amplification tests. *Vox Sanguinis.* 2003;85(1):1-8. |
| 12. | Kang JW, Seo JH, Youn KW, et al. Use of supplemental anti-HBc testing of donors showing non-discriminating reactive results in multiplex nucleic acid testing. *Vox Sang.* 2017;112(7):622-627. |
| 13. | Katsoulidou A, Moschidis Z, Sypsa V, et al. Analytical and clinical sensitivity of the Procleix Ultrio HIV-1/HCV/HBV assay in samples with a low viral load. *Vox Sang.* 2007;92(1):8-14. |
| 14. | Mafirakureva N, Mapako T, Khoza S, et al. Cost effectiveness of adding nucleic acid testing to hepatitis B, hepatitis C, and human immunodeficiency virus screening of blood donations in Zimbabwe. *Transfusion.* 2016;56(12):3101-3111. |
| 15. | Müller B, Nübling CM, Kress J, Roth WK, De Zolt S, Pichl L. How safe is safe: new human immunodeficiency virus Type 1 variants missed by nucleic acid testing. *Transfusion.* 2013;53(10 Pt 2):2422-2430. |
| 16. | Pruessmann JN, Langan EA, Rupp J, et al. Challenge of hepatitis B testing following intravenous immunoglobulin therapy in patients with autoimmune skin diseases. *J Dermatol.* 2022;49(10):1049-1051. |
| 17. | Servant-Delmas A, Chuteau C, Lefort C, et al. Two cases of transfusion-transmitted hepatitis B virus (HBV) infection in a low-endemic country before implementation of HBV nucleic acid testing. *Transfusion.* 2013;53(2):291-296. |
| 18. | Stramer SL, Zou S, Notari EP, et al. Blood donation screening for hepatitis B virus markers in the era of nucleic acid testing: are all tests of value? *Transfusion.* 2012;52(2):440-446. |
| 19. | Stramer SL, Krysztof DE, Brodsky JP, et al. Comparative analysis of triplex nucleic acid test assays in United States blood donors. *Transfusion.* 2013;53(10 Pt 2):2525-2537. |
| 20. | Valentine-Thon E. Borderline and/or discordant Cobas Amplicor HCV Test version 2.0 results: clinical significance. *J Clin Virol.* 2001;20(1-2):77-80. |
| 21. | Vargo J, Smith K, Knott C, et al. Clinical specificity and sensitivity of a blood screening assay for detection of HIV-1 and HCV RNA. *Transfusion.* 2002;42(7):876-885. |
| 22. | Weber B, Mühlbacher A, Melchior W. Detection of an acute asymptomatic HBsAg negative hepatitis B virus infection in a blood donor by HBV DNA testing. *J Clin Virol.* 2005;32(1):67-70. |
| 23. | Wesolowski LG, Delaney KP, Hart C, et al. Performance of an alternative laboratory-based algorithm for diagnosis of HIV infection utilizing a third generation immunoassay, a rapid HIV-1/HIV-2 differentiation test and a DNA or RNA-based nucleic acid amplification test in persons with established HIV-1 infection and blood donors. *J Clin Virol.* 2011;52 Suppl 1:S45-49. |
| 24. | Hasegawa I, Nakano T, Koguchi H, et al. Subclinical hepatitis E virus (HEV) infection detected by nucleic acid amplification test on blood donation: short-term positivity for immunoglobulin G class of antibody against HEV. *Clin J Gastroenterol.* 2022;15(4):750-754. |
| 25. | Porto-Ferreira FA, de Almeida-Neto C, Murphy EL, et al. A randomized trial to evaluate the use of text messaging, letter, and telephone call reminders to improve return of blood donors with reactive serologic tests. *Transfusion.* 2017;57(1):102-107. |
| 26. | Vermeulen M, Van Drimmelen H, Coleman C, Mitchel J, Reddy R, Lelie N. A mathematical approach to estimate the efficacy of individual-donation and minipool nucleic acid amplification test options in preventing transmission risk by window period and occult hepatitis B virus infections. *Transfusion.* 2014;54(10):2496-2504. |
| 27. | Grabarczyk P, van Drimmelen H, Kopacz A, et al. Head-to-head comparison of two transcription-mediated amplification assay versions for detection of hepatitis B virus, hepatitis C virus, and human immunodeficiency virus Type 1 in blood donors. *Transfusion.* 2013;53(10):2512-2524. |
| 28. | Huang WL, Liao YH, Lin YJ, Wei ST, Hou SM, Yang JY. Investigation of transfusion associated hepatitis C virus infection in Taiwan, 2015–2018. *Journal of the Formosan Medical Association.* 2020;119(3):752-756. |
| 29. | Zbinden A, Ries J, Redli PM, et al. Prevalence of Occult Hepatitis B Virus Infection in Blood Donors with Negative ID-NAT in Switzerland. *Transfusion Medicine and Hemotherapy.* 2022;49(6):338-345. |
| 30. | Qiu X, Sokoll L, Duong Ly T, et al. An improved HIV antigen/antibody prototype assay for earlier detection of acute HIV infection. *Journal of Clinical Virology.* 2021;145. |
| 31. | Berg MG, Olivo A, Harris BJ, et al. A high prevalence of potential HIV elite controllers identified over 30 years in Democratic Republic of Congo. *EBioMedicine.* 2021;65. |
| 32. | Patel J, Jansari TR, Chauhan A. Retrospective study of blood transfusion transmitted infections (Hiv, hcv, hbv, syphilis & malaria] among the blood donors in Dhiraj hospital. *Medico-Legal Update.* 2021;21(2):663-668. |
| 33. | Lee EJ, Kwon SY, Oh KM, et al. High risk donors management based on the results of HIV-1 NAT screening test. *Vox Sanguinis.* 2014;107:135-136. |
| 34. | Palmeira MK, Hermes RB, Lemos JR, et al. The experience of hemotherapy and hematology center of para-hemopa with nucleic acid amplification testing (NAT): First detection of human immunodeficiency virus (HIV) in window period. *Transfusion.* 2013;53:210A-211A. |
| 35. | Zhang R, Sun Y, Wang L, Zhang K, Xie J, Li J. Blood screening for human immunodeficiency virus: A new algorithm to reduce the false-positive results. *Transfusion Medicine.* 2013;23(4):260-264. |
| 36. | Meyer T, Polywka S, Wulff B, et al. Virus NAT for HIV, HBV, and HCV in post-mortal blood specimens over 48 h after death of infected patients - First results. *Transfusion Medicine and Hemotherapy.* 2012;39(6):376-380. |
| 37. | Contreras AM, Reta CB, Torres O, Celis A, Domínguez J. [Safe blood in the absence of viral infections due to HBV, HCV and HIV in serological window period in donors]. *Salud pública de México.* 2011;53 Suppl 1:S13-18. |
| 38. | Bingulac-Popovic J, Dogic V, Babic I, Sarlija D, Balija M, Jukic I. Evaluation study of new commercial Sacace triplextest for detection of HBV-DNA, HCV-RNAAND HIV-RNA. *Vox Sanguinis.* 2011;101:197. |
| 39. | Byrne L, Brant LJ, Davison K, Hewitt P. Transfusion-transmitted human immunodeficiency virus (HIV) from seroconverting donors is rare in England and Wales: Results from HIV lookback, October 1995 through December 2008. *Transfusion.* 2011;51(6):1339-1345. |
| 40. | Bamaga MS, Bokhari FF, Aboud AM, Al-Malki M, Alenzi FQ. Nucleic acid amplification technology screening for hepatitis C virus and human immunodeficiency virus for blood donations. *Saudi Medical Journal.* 2006;27(6):781-787. |
| 41. | Behzad-Behbahani A, Mafi-Nejad A, Tabei SZ, Lankarani KB, Torab A, Moaddeb A. Anti-HBc & HBV-DNA detection in blood donors negative for hepatitis B virus surface antigen in reducing risk of transfusion associated HBV infection. *Indian Journal of Medical Research.* 2006;123(1):37-42. |
| 42. | Ramia S, Ramlawi F, Kanaan M, Klayme S, Naman R. Frequency and significance of antibodies against hepatitis B core (anti-HBc) antigen as the only serological marker for hepatitis B infection in Lebanese blood donors. *Epidemiology and Infection.* 2005;133(4):695-699. |
| 43. | Fabrizi F, de Vecchi AF, Como G, Lunghi G, Martin P. De novo HCV infection among dialysis patients: a prospective study by HCV core antigen ELISA assay. *Alimentary Pharmacology & Therapeutics.* 2005;21(7):861-869. |
| 44. | Ries J, Frey BM, Gottschalk J, et al. Occult hepatitis B virus infection in blood donors missed by standard hepatitis B screening. *Transfusion Medicine and Hemotherapy.* 2018;45:31-32. |
| 45. | De Zolt S, Thermann R, Bangsow T, et al. Implementation of an HIV-1 Triple-Target NAT Assay in the Routine Screening at Three German Red Cross Blood Centres. *Transfusion Medicine and Hemotherapy.* 2016;43(3):183-189. |
| 46. | Alvarado-Mora MV, Botelho L, Nishiya A, et al. Frequency and genotypic distribution of GB virus C (GBV-C) among Colombian population with Hepatitis B (HBV) or Hepatitis C (HCV) infection. *Virology Journal.* 2011;8. |
| 47. | Zubkova NV, Filatova EV, Zubov SV. Serological and molecular genetic markers of hepatitis C virus in infected donors. *Voprosy Virusologii.* 2010;55(5):34-36. |
| 48. | Abou-Raya A, Abou-Raya S, Helmii M. Prevalence of antibodies against hepatitis C virus in patients with systemic lupus erythematosus. *Lupus.* 2010;19:126-127. |
| 49. | Gadano A, Galdame O, Marciano S. Diagnosis of patients with suspected chronic hepatitis C infection. *Annals of Hepatology.* 2010;9(SUPPL. 1):34-38. |
| 50. | Furuta RA, Kondo Y, Saito T, et al. Transfusions of red blood cells from an occult hepatitis B virus carrier without apparent signs of transfusion-transmitted hepatitis B infection. *Transfusion Medicine.* 2008;18(6):379-381. |
| 51. | Rashdan A, Hijjawi S, Jadallah K, Matalka I. Prevalence of hepatitis C virus antibodies among blood donors in Northern Jordan. *Jordan Medical Journal.* 2008;42(3):179-183. |
| 52. | Wiedmann M, Kluwick S, Walter M, et al. HIV-1, HCV and HBV seronegative window reduction by the new Roche cobas® TaqScreen MPX test in seroconverting donors. *Journal of Clinical Virology.* 2007;39(4):282-287. |
| 53. | Fabrizi F, De Vecchi AF, Como G, Lunghi G, Martin P. De novo HCV infection among dialysis patients: A prospective study by HCV core antigen ELISA assay. *Alimentary Pharmacology and Therapeutics.* 2005;21(7):861-869. |
| 54. | Teixeira SLM, Bastos FI, Telles PR, et al. HIV-1 infection among injection and ex-injection drug users from Rio de Janeiro, Brazil: Prevalence, estimated incidence and genetic diversity. *Journal of Clinical Virology.* 2004;31(3):221-226. |
| 55. | Mine H, Emura H, Miyamoto M, et al. High throughput screening of 16 million serologically negative blood donors for hepatitis B virus, hepatitis C virus and human immunodeficiency virus type-1 by nucleic acid amplification testing with specific and sensitive multiplex reagent in Japan. *Journal of Virological Methods.* 2003;112(1-2):145-151. |
| 56. | Gonçales Jr FL, Pereira JSF, Da Silva C, et al. Hepatitis B virus DNA in sera of blood donors and of patients infected with hepatitis C virus and human immunodeficiency virus. *Clinical and Diagnostic Laboratory Immunology.* 2003;10(4):718-720. |
| 57. | Liu CJ, Chen PJ, Shau WY, Kao JH, Lai MY, Chen DS. Clinical aspects and outcomes of volunteer blood donors testing positive for hepatitis-C virus infection in Taiwan: A prospective study. *Liver International.* 2003;23(3):148-155. |
| 58. | Candotti D, Richetin A, Cant B, et al. Evaluation of a transcription-mediated amplification-based HCV and HIV-1 RNA duplex assay for screening individual blood donations: A comparison with a minipool testing system. *Transfusion.* 2003;43(2):215-225. |
| 59. | Muller Z, Deak J, Horanyi M, et al. The detection of hepatitis C virus in South Hungary. *Journal of Clinical Virology.* 2001;20(1-2):81-83. |
| 60. | Cardoso MS, Koerner K, Hinz W, et al. Experiences in HCV-NAT screening prior to releasing cellular components by the German Red Cross Blood Transfusion Service of Baden-Wurttemberg. *Biologicals.* 1999;27(4):281-284. |
