# Supplementary material for: Improved Safety of Nucleic Acid Amplification Technology Combined With Serological Tests for Screening Blood Donors: A Systematic Review and Meta‐Analysis
Source: Rev Med Virol. 2026 Feb 21;36(2):e70117. doi: 10.1002/rmv.70117 (PMC12924692; doi:10.1002/rmv.70117)
Supplement: Supplementary file 4 — Supporting Information S4 [file RMV-36-e70117-s001.docx]

SUPPLEMENTARY FILE 4 Funnel plot of the meta-analysis of the frequency of concordance of negative serological tests and NAT positive for HBV concerning the total number of donors according to random effects analysis

SUPPLEMENTARY FILE 4 Funnel plot of the meta-analysis of the frequency of concordance of negative serological tests and NAT positive for HCV concerning the total number of donors according to random effects analysis

SUPPLEMENTARY FILE 4 Funnel plot of the meta-analysis of the frequency of concordance of negative serological tests and NAT positive for HIV concerning the total number of donors according to random effects analysis
