# Supplementary material for: Improved Safety of Nucleic Acid Amplification Technology Combined With Serological Tests for Screening Blood Donors: A Systematic Review and Meta‐Analysis
Source: Rev Med Virol. 2026 Feb 21;36(2):e70117. doi: 10.1002/rmv.70117 (PMC12924692; doi:10.1002/rmv.70117)
Supplement: Supplementary file 5 — Supporting Information S5 [file RMV-36-e70117-s005.docx]

SUPPLEMENTARY FILE 5 Funnel plot of Meta-analysis of the frequency of retrieval of HBV positive NAT test concordance after negative serology donors

SUPPLEMENTARY FILE 5 Funnel plot of Meta-analysis of the frequency of retrieval of HCV positive NAT test concordance after negative serology donors

SUPPLEMENTARY FILE 5 Funnel plot of Meta-analysis of the frequency of retrieval of HIV positive NAT test concordance after negative serology donors
